# Reactivity Properties of Mixed- and High-Valent Bis(μ-Hydroxide)-Dinickel Complexes

**DOI:** 10.1021/acsomega.1c04225

**Published:** 2021-10-15

**Authors:** Giuseppe Spedalotto, Marta Lovisari, Aidan R. McDonald

**Affiliations:** School of Chemistry, Trinity College Dublin, The University of Dublin, College Green, Dublin 2, Ireland

## Abstract

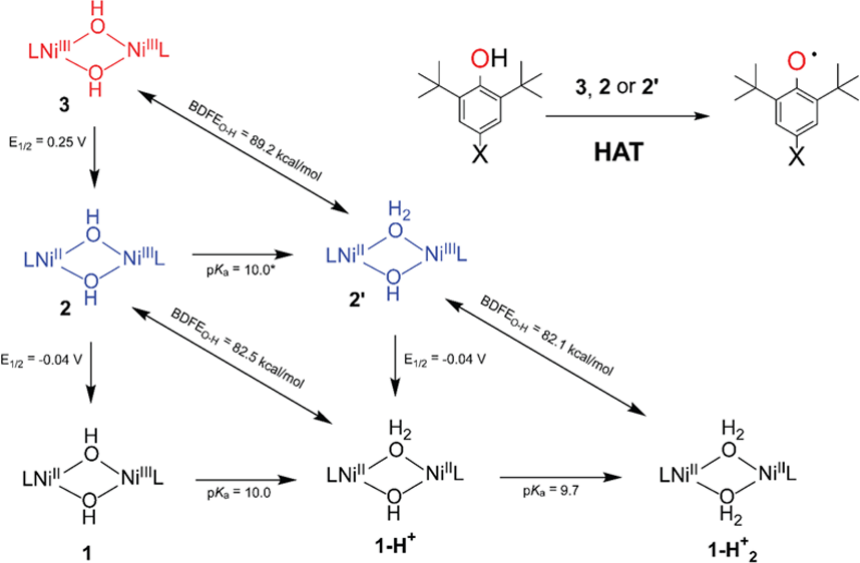

Despite their potential role in enzymatic systems, there is a dearth of hydroxide-bridged high-valent oxidants. We recently reported the synthesis and characterization of Ni^II^Ni^III^(μ-OH)_2_ (**2**) and Ni_2_^III^(μ-OH)_2_ (**3**) species supported by a dicarboxamidate ligand (*N*,*N*′-bis(2,6-dimethyl-phenyl)-2,2-dimethylmalonamide). Herein, we explore the oxidative reactivity of these species using a series of para-substituted 2,6-di-*tert*-butyl-phenols (4-X-2,6-DTBP, X = −OCH_3_, −CH_2_CH_3_, −CH_3_, −C(CH_3_)_3_, −H, −Br, −CN, and −NO_2_) as a mechanistic probe. Interestingly, upon reaction of **3** with the substrates, the formation of a new transient species, **2′,** was observed. **2′** is postulated to be a protic congener of **2**. All three species were demonstrated to react with the substituted phenols through a hydrogen atom transfer reaction mechanism, which was elucidated further by analysis of the postreaction mixtures. Critically, **3** was demonstrated to react at far superior rates to **2** and **2′**, and oxidized substrates more efficiently than any bis-μ-oxo-Ni_2_^III^ reported to date. The kinetic superiority of **3** with respect to **2** and **2′** was attributed to a stronger bond in the product of oxidation by **3** when compared to those calculated for **2** and **2′**.

## Introduction

Small-molecule activation is a large class of chemical transformations, which are considered the pillars of the modern chemical industry.^1−3^ Among them, the catalytic oxidative activation of C–H bonds present in saturated hydrocarbons is considered one of the most important modern challenges^4^ because hydrocarbons constitute the most abundant and utilized feedstock for small-molecule chemical industries. However, this class of compounds exhibits an overall inertness to functionalization due to the strength of their C–H bonds (bond dissociation enthalpy (BDE_C–H_) = 75–105 kcal/mol),^5^ requiring unsustainable processes (elevated temperature, high pressure, and low selectivity) for their conversion to more valuable and reactive refined products.^6^ Metalloenzymes such as oxygenases are able to activate atmospheric dioxygen to oxidize strong C–H bonds under physiological conditions. The development of biomimetic catalysts, synthetically accessible and capable of performing similar processes to their enzymatic counterparts, is, therefore, a promising path to pursue.

Dinuclear high-valent metal–oxygen adducts have been postulated to be the active oxidants in several of the most impressive oxygenase enzymes.^7,8^ These include tyrosinase and the soluble methane monooxygenase (sMMO), notably one of the few enzymes able to oxidatively convert methane into methanol.^8,9^ While the structure and reactivity of the tyrosinases have been widely studied, identifying the oxy-Ty and met-Ty (i.e., the oxygenated, Cu^II^_2_(μ-O_2_), and the metastable, Cu^II^_2_(μ-OH) forms of the tyrosinase active site) as the reactive intermediates,^10−13^ the structure of the di-iron-active intermediate of the sMMO has been questioned, suggesting that a hydroxide-bridged di-iron adduct and not the originally postulated bis-μ-oxo-Fe^IV^_2_ could be the active oxidant in the activation of CH_4_.^14^ These pieces of experimental evidence suggest that dinuclear μ-hydroxide complexes may play a more important role than previously thought. Indeed, it is not unrealistic to hypothesize that, in an aqueous environment such as the physiological one in which the enzymes operate, the main high-valent species generally identified as μ-oxo or μ-peroxo could exist, even if transiently, as protonated species. Moreover, these species might be the active oxidant or possibly intermediates formed upon an initial hydrogen atom abstraction event. Therefore, the analysis of the role of such systems will be fundamental to have a complete picture of the mechanism involved and, consequently, mimic the enzymatic behavior on a synthetic scale.

**Scheme 1:**
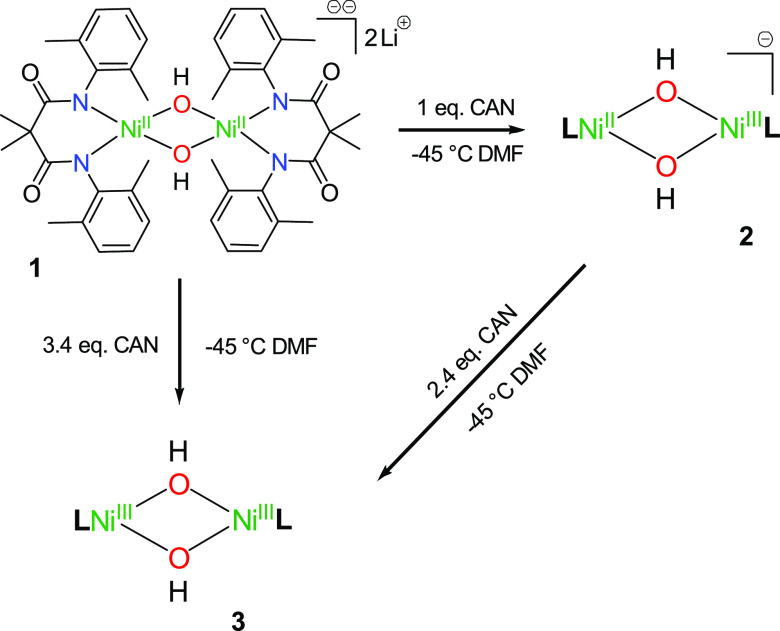
Preparation of **2** and **3** by Oxidation of **1**(18) L = *N*,*N*′-bis(2,6-dimethyl-phenyl)-2,2-dimethylmalonamidate.

Despite a plethora of dinuclear oxo-bridged systems,^15−17^ a dearth of synthetic hydroxide bridged high-valent oxidants is present in the literature, especially for late transition metals such as Co, Ni, and Cu. Recently, we reported the first example of high-valent hydroxide-bridged Ni^II^Ni^III^ and Ni^III^Ni^III^ complexes (**2** and **3**, Scheme 1) supported by a dianionic donor ligand L (*N*,*N*′-bis(2,6-dimethyl-phenyl)-2,2-dimethylmalonamidate).^18^ In the present work, we report a study of the reactivity properties of **2** and **3** toward a series of para-substituted 2,6-di-*tert*-butyl-phenols, widely used mechanistic probes for proton-coupled electron transfer (PCET) oxidation reactions,^19−22^ with the aim of understanding the mechanism and driving forces through which the these high-valent hydroxide-bridged species perform PCET.

## Results

The bis-μ-hydroxide mixed-valent species **2** and the high-valent species **3** (Scheme 1) were prepared according to the procedure reported in our previous work.^18^ Mixed-valent Ni^II^Ni^III^**2** was prepared by a reaction of **1** with one equivalent of CAN, while Ni^III^_2_**3** was prepared by a reaction of **1** with a total of 3.4 equivalent of CAN.

**Figure 1:**
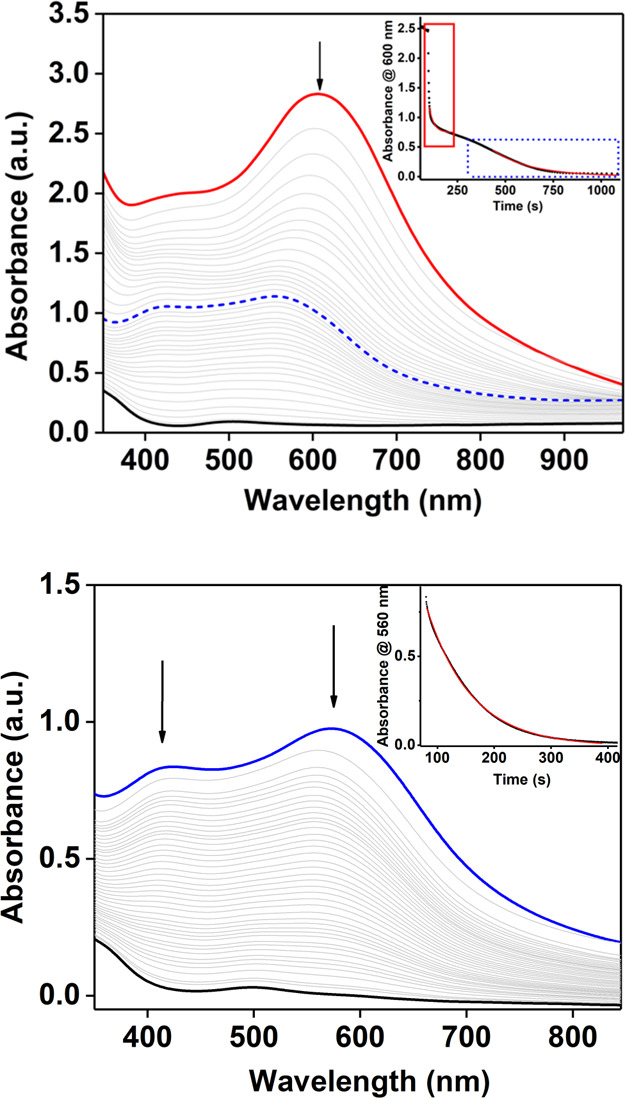
Top. Electronic absorption spectrum showing the decay of **3** (red trace) upon reaction with 4-CH_3_-2,6-DTBP (50 equiv) and the formation of **2′** (blue-dashed trace). Reaction performed at −45 °C in DMF. Top inset: decay of the λ = 600 nm feature of **3** upon addition of the substrate, showing the biphasic behavior. Bottom: the electronic absorption spectrum showing the decay of **2** (blue trace) upon reaction with 4-CH_3_-2,6-DTBP (50 equiv.) at −45 °C in DMF. Bottom inset: decay of λ = 560 nm feature of **2** against time.

**2** and **3** were reacted with a series of para-substituted 2,6-di-*tert*-butyl-phenols (4-X-2,6-DTBP, X = −OCH_3_, −CH_2_CH_3_, −CH_3_, −C(CH_3_)_3_, −H, −Br, −CN, and −NO_2_). The kinetics of the reactions were studied by electronic absorption spectroscopy, monitoring the change in the absorbance feature at λ = 600 nm for **3** and λ = 560 nm for **2** (Figure 1). The decay of each species upon reaction with the substrate was fitted with a pseudo-first-order kinetic model (substrates were added in >10 equiv excess), yielding a rate of decay (*k*_obs_). *k*_obs_ obtained displayed a linear dependency on [4-X-2,6-DTBP], allowing for the determination of the second-order reaction rate constant (*k*_2_) as the slope of the plot of *k*_obs_ against [4-X-2,6-DTBP].

An important observation was that upon reaction of **3** with an external substrate, the formation of a transient species with spectral features similar to those of **2** (species defined as **2′**) was observed (Figures 1 and S1), generating a biphasic decay pattern (Figure 1, inset). Notably, **2′** decayed at a significantly lower rate than **3** (after **3** had decayed). The kinetic data reported for **3**, therefore, refers only to the first phase of decay. The decay of **2′** (i.e., the second phase of the decay of **3**) was also analyzed, in order to fully understand the reaction mechanism involved. Electron paramagnetic resonance (EPR) analysis of species **2′** was consistent with the presence of an *S* = 1/2 metal-centered radical species (Figure S2, *g*_x_ = 2.45, *g*_y_ = 2.34, and *g*_z_ = 2.00; *g*_av_ = 2.26). Comparison with the reported EPR spectrum of the bis-μ-hydroxide Ni^II^Ni^III^ species, **2** revealed little-to-no differences (Figure S2),^18^ suggesting that the two species share a similar electronic and structural configuration, possibly displaying different degrees of protonation. We have been unable to prove conclusively that the species formed after oxidation by **3** is a new species (**2′**) or is simply complex **2**, the spectral data for both **2** and **2′** are remarkably similar. Attempts to independently prepare **2′** through protonation of **2** have resulted in the decomposition of the complex (details below). Given that **2′** forms after PCET oxidation by **3**, we have surmised that the new species is a protonated form of **2** (i.e., **2′**).

### Reactivity of 3

**3** reacted readily with all the 4-X-2,6-DTBP substrates at −45 °C in DMF (Table 1, Figures S3–S9). Reaction of **3** with the 4-X-2,6-DTBP substrates, where X = OCH_3_, C(CH_3_)_3_ led to the formation of the corresponding phenoxyl radicals (X = OCH_3_, λ = 408, 390 nm; X = C(CH_3_)_3_, λ = 403, 384 nm, Figure S11).^23−25^ Comparison with the corresponding independently synthesized phenoxyl radicals confirmed the formation of the phenoxyl radical (for X = OCH_3_, λ = 407, 390 nm, Figure S14; for X = C(CH_3_)_3_, λ = 402, 384 nm, Figure S15). The identity of these species was further confirmed by EPR spectroscopy (Figures S12–S13). Reaction of **3** with the substrates X = CH_2_CH_3_ and CH_3_ resulted in featureless spectra in the visible region (Figure S11). Therefore, the products for these substrates were detected by electro-spray ionization (ESI) mass spectrometry (Figure S16) and identified as 2,6-di-*tert*-butyl-4-ethylidene-2,5-cyclohexadienone (X = CH_2_CH_3_) and 2,6-di-*tert*-butyl-4-methylene-2,5-cyclohexadienone (X = CH_3_). Reaction of **3** with substrates X = H and X = Br resulted in a common spectrum with features at λ = 430 and 408 nm (Figure S11), consistent with the formation of the coupled product 3,3′,5,5′-tetra-*tert*-butyl-4,4′-diphenoquinone (DPQ, Figure S17). The formation of the diphenoquinone is a widely reported outcome in the case of hydrogen atom transfer oxidation for X = H.^26,27^ The presence of this product for X = H/Br can be assigned to the formation of an unstable phenoxy radical upon a PCET event, followed by dimerization to form the diphenyl quinone.

**Table 1:** *k*_2_-Values and End Reaction Products for the Reactions of **3**, **2**, and **2′** with 4-X-2,6-DTBP at −45 °C in DMF

	**3**			**2**			**2′**^c^
x	*k*_2_ (M^–1^ s^–1^)	products	yield^a^%	*k*_2_ (M^–1^ s^–1^)	Products	Yield^a^ %	*k*_2_ (M^–1^ s^–1^)
–OCH_3_	520(11)	phenoxyl radical	35 ± 10	84.6(3)	phenoxyl radical	66 ± 6 (49 ± 20)^b^	41(1)
–CH_2_CH_3_^d^	29(1)	*p*-C_2_H_4_-CD		0.84(2)	*p*-C_2_H_4_-CD		0.553(3)
–CH_3_ [H]^d^	24.7(11)	*p*-CH_2_-CD		0.76(4)	*p*-CH_2_-CD		0.67(1)
–CH_3_ [D]	5.8(5)^18^	*p*-CH_2_-CD^18^		0.37(3)	*p*-CH_2_-CD		0.140(4)
	KIE= 4			KIE = 2			KIE = 4.8
–C(CH_3_)3	22(1)	phenoxyl radical	52 ± 27	0.45(1)	phenoxyl radical	88 ± 14 (64 ± 20)^b^	0.42(1)
–H	1.16(3)	DPQ	10 ± 2	0.045(2)	DPQ	11 ± 2	0.042(2)
–Br	1.28(4)	DPQ	6 ± 1	0.031(3)	DPQ	15 ± 2	0.08(1)
–CN				0.99(1)	DPQ	18 ± 2	
–NO_2_	52.0(24)	4-NO_2_-2,6-DTB phenoxide		0.65(3)	4-NO_2_-2,6-DTB phenoxide		0.45(2)

a Yields estimated by UV–vis considering the amount of Ni^III^ in solution.

b Radical yield estimated by double integration of the EPR spectrum. Abbreviations: *p*-C_2_H_4_-CD = 2,6-di-*tert*-butyl-4-ethylidene-2,5-cyclohexadienone; *p*–CH_2_–CD = 2,6-di-*tert*-butyl-4-methylene-2,5-cyclohexadienone; and DPQ = 3,3′,5,5′-tetra-*tert*-butyl-4,4′-diphenoquinone.

c Not possible to accurately determine products and yields for **2′**(because **3** was also active in the same mixture).

d For these substrates, it was not possible to accurately quantify product yields.

For X = NO_2_, the reaction displayed an initial rapid decay of **3**, followed by the formation of a new species, which rapidly saturated the spectrum, making its identification non-trivial (Figure S18). Neither formation of **2′**, nor evidence of oxidation events (e.g., formation of radical species or DPQ) were observed. Considering the features that grew-in (λ = 452 and 466 nm) before saturation of the spectrum, the product was identified as the 4-NO_2_-2,6-di-*tert*-butyl-phenolate anion by comparison with a solution of 4-NO_2_-2,6-di-*tert*-butyl sodium phenoxide independently prepared (Figure S19). Unfortunately, the reaction between **3** and 4-CN-2,6-DTBP exhibited an unexpected trend (Figure S10), with an initial increase of the absorbance at λ = 600 nm, followed by a partial decay. This prevented us from interpreting the decay with a pseudo-first-order kinetic model. We tentatively suggest that the nitrile group in 4-CN-2,6-DTBP either coordinated to the Ni_2_^III^ core or suffered from nucleophilic attack by the bridging hydroxide ligands, resulting in an alternative decay pathway. The reactivity of **3** with 4-NO_2_/CN-2,6-DTBP will thus be considered as outliers and will not be used in our mechanistic analyses. Overall, for all substrates except X = CN and NO_2_, product analysis suggested that the initial PCET oxidation of the phenol substrates to yield phenoxyl radicals occurred, followed by further decay to some of the identified products.

Quantification of the end reaction products was performed by UV–vis and EPR spectroscopies (Table 1). Species **3** can perform PCET on the substrates giving **2′** as the product, which further reacted with excess substrate. In the case of substrates generating stable phenoxyl radicals (X = OCH_3_, C(CH_3_)_3_), the oxidation yields of these substrates were estimated as the difference between the overall moles of phenoxyl radical detected at the end of the reaction of **3** and the moles of radical due to **2’**. Considering the transient nature of **2′**, we postulated that the amount of radical generated by reaction with **2′** would match the one obtained for **2** (see below). The conversion yields for the formation of the radical species, calculated with respect to the moles of Ni^III^ present in solution, were consistent with a 1e^–^ oxidation mechanism by **3**. For X = CH_3_ and C_2_H_5_, it was not possible to quantify the yields of the products that were identified using ESI-MS. To have a reliable quantification of the products generated by **3** for substrates that did not yield an identifiable phenoxyl radical (X = H, Br), we quenched the reaction after **3** had decayed (by addition of trifluoracetic acid, TFA) but prior to the decay of **2′**. The low yields of DPQ observed for X = H and Br were not unexpected because the observed product DPQ was the result of dimerization of the metastable phenoxyl radical, which could easily undergo different decay pathways.^28−30^ Such observations were again consistent and coherent with a PCET oxidation mechanism.

To explore the fate of the Ni complex after substrate oxidation, ^1^H NMR analysis of the final reaction mixture for the reaction of **3** with 4-OCH_3_-2,6-DTBP in deuterated DMF was performed (Figure S20). The resonance of the bridging −OH ligands, found at δ = −9.22 ppm for **1**, was not present, while the two C*H*_3_ resonances of the ligand backbone in the complex at δ = 2.08 ppm and δ = 2.36 were shifted to values typical of the free ligand, δ = 1.78 ppm and δ = 2.24 ppm, respectively. Moreover, a peak at δ = 9.14 ppm corresponding to the resonance of the −NH groups of the protonated ligand ^Me_2_^DMMAH_2_ was observed. A broadening and a shift of the H_2_O peak from δ = 3.47 ppm to δ = 3.12 ppm was also observed, suggesting a change of pH in solution or the establishment of a new hydrogen bond interaction, presumably as a result of PCET oxidation by the complexes yielding an increased H^+^ concentration in solution.^31,32^ This evidence suggests that a total consumption of the high-valent species **3** and **2′** had occurred and their conversion to the free ligand and presumably free Ni^II^ ions. Finally, the characteristic set of peaks of 2,6-di-*tert*-butyl-1,4-benzoquinone at δ = 1.29 ppm and δ = 6.58 ppm suggested its formation in solution, identifying it as the final(over)oxidation decay product of the previously observed phenoxyl radical (Table 1).

### Reactivity of **2**

**2** also reacted readily, albeit at lower rates, with the selected 4-X-2,6-DTBP substrates at −45 °C in DMF (Table 1, Figures S21–S28). Analysis of the postreaction mixtures for this set of reactions identified the same oxidation products previously observed for **3** (Table 1, Figure S29–S33), suggesting a common oxidation mechanism (i.e., PCET). However, in contrast to **3**, it was possible to analyze the reaction of **2** with X = CN, which resulted in the formation of DPQ as the product. Analogous to X = H, Br, the formation of the diphenyl quinone for X = CN was attributed to the dimerization of the unstable phenoxy radical formed upon the above proposed PCET mechanism. Furthermore, the reaction of **2** with X = NO_2_ resulted again in the rapid decay of the oxidant, followed by the formation of 4-NO_2_-2,6-di-*tert*-butyl-phenolate (Figure S33). No evidence of substrate oxidation was observed, and X = NO_2_ was thus considered an outlier for our kinetic analysis below.

The conversion yields calculated upon quantification of the products were consistent with a 1e^–^ oxidation event, in particular for the radical-generating substrates X = CH_3_ and OCH_3_ (Table 1). As previously observed for **3**, substrates with X = H, Br, and CN exhibited low conversion yields (Table 1) because the formation of the UV–Vis silent 2,6-di-*tert*-butyl-1,4-benzoquinone is competitive with the formation of the analyzed DPQ.

^1^H NMR analysis of the final reaction mixture from the reaction of **2** with 4-OCH_3_-2,6-DTBP in deuterated DMF (Figure S34) appeared to be consistent with the total conversion of the complex **2** to the free ligand (already observed in the case of **3**/**2′**), given the presence of the characteristic resonance peaks of the protonated ligand, such as the −NH groups at peak at δ = 9.14 ppm. A broadening and a shift of the H_2_O peak (from δ = 3.47 ppm to δ = 3.28 ppm), consistent with a variation of the pH/hydrogen bonding network in solution, was observed. Furthermore, the presence of the characteristic set of peaks of 2,6-di-*tert*-butyl-1,4-benzoquinone as an oxidation product of the 4-OCH_3_-2,6-DTBP was coherent with the PCET mechanism postulated by the analysis of the products and already observed above for species **3**.

### Reactivity of 2′

**Scheme 2:**
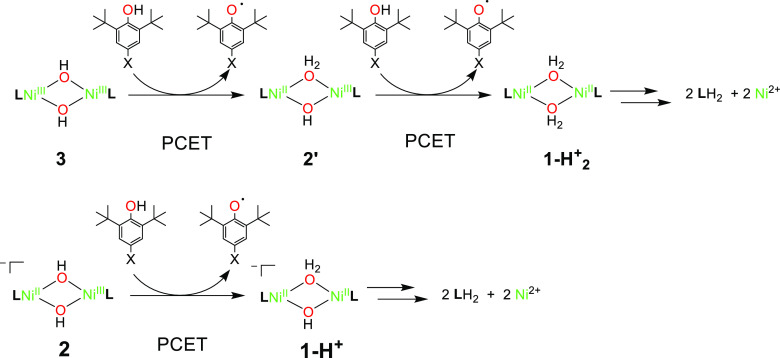
Proposed Reaction Mechanism for the Reaction of **3**, **2′** and **2** with Substrates

**2′** is proposed to form from the PCET reaction of **3** with a substrate, it is thus postulated to contain a Ni^II^Ni^III^(μ-OH)(μ-OH_2_) core (Scheme 2). The reactivity studies of **2′** with the selected substrates were performed contemporaneously to the reactivity of **3** (Table 1, Figures S35–S40). However, a proper analysis and quantification of the end reaction products of the oxidation of the substrates by **2′** was not possible due to the presence of products already generated by **3**.

In our previous work, we reacted species **3** and **2** with the deuterated 4-CH_3_-[*D*]-2,6-DTBP, obtaining the corresponding kinetic isotope effect values (KIE ∼4 for **3** and KIE ∼ 2 for **2**, Table 1).^18^ Similarly, in the present work, the reaction of **2′** with the deuterated 4-CH_3_-[*D*]-2,6-DTBP was performed (Figure S41), obtaining a measured KIE value with respect to the couple 4-CH_3_-[*H*]/[*D*]-2,6-DTBP of 4.8 (Table 1, Figure S42). This result suggested the presence of a hydrogen atom/proton transfer event in the rate-determining step.

### Probing the Mechanism of PCET

We propose a reaction mechanism for the oxidation of phenols by **3**, **2′**, and **2** by PCET (Scheme 2), where, in the PCET oxidation of substrates, **3** is converted to **2′**, **2** is converted to **1-H**^**+**^, and **2′** is converted to **1-H**_**2**_^**+**^. Postreaction ^1^H NMR experiments showed the formation of a free protonated ligand, indicating that **1-H**^**+**^ and **1-H**_**2**_^**+**^ are likely unstable and decayed presumably via ligand protonation by the newly formed H_2_O ligands.

In an attempt to understand the driving forces for oxidations by **3**, **2**, and **2′**, we explored the preparation of protonated adducts of **1** (i.e., **1-H**^**+**^ and **1-H**^**+**^_**2**_) and **2** (**2-H**^**+**^), which are postulated to be the products of oxidation by **2**, **2′**, and **3**, respectively (Scheme 2), and thus provide the driving force for PCET oxidation. Different weak organic acids were preliminarily screened for reaction with **1** under the oxidation reaction conditions (−45 °C in DMF, Table S1, Figures S43–S45), and substituted benzoic acids were identified to show an immediate reaction, accompanied by an 8–38 nm range red shift of the d–d band of **1** at λ = 502 nm (Figure S45). 2,6-dimethyl-benzoic acid yielded a stable species, probably due to the role of the sterically hindered benzoate counterion. In the reaction between **2** and a relatively weak acid (2,6-dimethylbenzoic acid), no change in the spectrum was observed (Figures S46). Such an observation was ambiguous because it could be indicative of both the absence of reaction between **2** and **2′** or conversion of **2** to **2′** because they share the exact same spectral features (see above) and cannot be currently distinguished. In the reaction between **2** and a stronger acid (pyridinium triflate), we observed bleaching of the features associated with **2** (Figure S47) and no new species that could be attributed to a protonated adduct (**2′**). We thus conclude that **1** is readily protonated with carboxylic acids, while it is currently not possible to obtain insights into changes in the protonation state of **2**.

A solution containing **1** and 2,6-dimethylbenzoic acid (4 equiv) was treated with 1,8-diazabicyclo(5.4.0)undec-7-ene (DBU, 4 equiv, Figure S48), causing the restoration of the feature of **1** at λ = 502 nm (Figure S48). Addition of CAN (3.4 equiv) to this solution led to the formation of **3** in similar yields to our original preparations of **3**, suggesting that the concentration of **1** was reverted to its initial extent upon addition of the base, DBU. Such observations indicated the presence of an equilibrium between **1** and protonated species in the presence of 2,6-dimethyl-benzoic acid.

**Figure 2:**
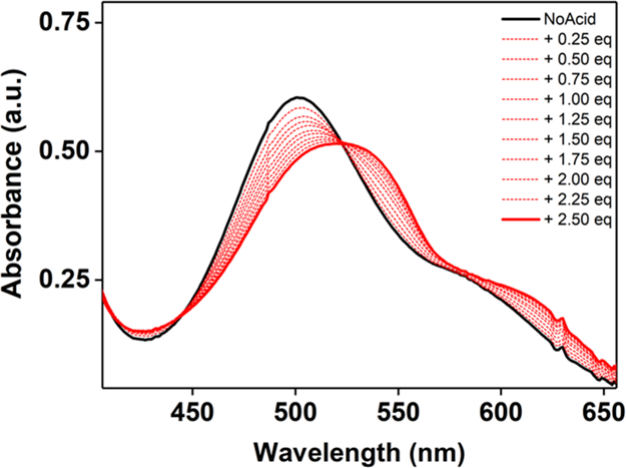
UV–vis monitored titration of **1** (black trace) with substoichiometric amounts of 2,6-dimethylbenzoic acid at −45 °C in DMF. Each dashed line corresponds to an increment of 0.25 equiv to a maximum of 2.5 equiv.

**Figure 3:**
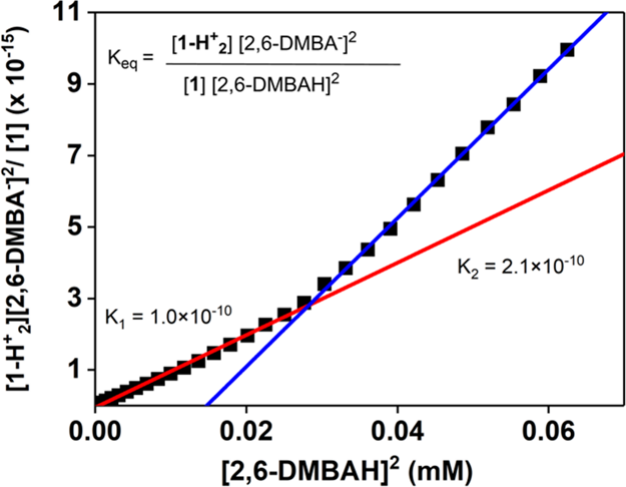
Plot of [2,6-DMBAH]^2^ vs [**1-H**^**+**^_**2**_][2,6-DMBA^–^]^2^/[**1**]. 2,6-DMBAH = 2,6-dimethyl benzoic acid; 2,6-DMBA^–^ = 2,6-dimethyl-benzoate.

Therefore, we performed an electronic absorption spectroscopy monitored titration of **1** with 2,6-dimethyl-benzoic acid (Figures 2, S49). Up to a maximum of 2.5 equiv of acid, we observed a clear conversion of **1** to a different species with λ_max_ = 535 nm. Addition of further equivalents of acid caused a progressive decay of the intensity of this feature, probably due to the instability of protonated species (**1-H**^**+**^ and **1-H**^**+**^_**2**_), albeit no change in λ_max_ was observed. A plot of [**1-H**^**+**^_**2**_][2,6 DMBA^–^]^2^/[**1**] vs [2,6 DMBAH]^2^ (where [**1-H**^**+**^_**2**_] represents the concentration of protic adducts in solution) indicated that **1** was protonated two times by the 2,6-dimethyl-benzoic acid (Figure 3). Attempts to fit the data as a single-protonation event resulted in non-linear graphs (Figures S50). The obtained plot was best fit with two separate linear trends (Figure 3), suggesting that the protonation reaction was not a simple A-to-B conversion,^33^ but a stepwise A-to-B-to-C reaction, namely the stepwise protonation of the hydroxide groups on **1** to form the monoprotonated **1-H**^**+**^ and subsequently the diprotonated **1-H**^**+**^_**2**_. The slope of the fits, which correspond to the equilibrium constants of the two reactions, has been calculated to be *K*_1_ = 1.0 × 10^–10^ and *K*_2_ = 2.1 × 10^–10^ (Figure 3). Therefore, the derived p*K*_a_ values of the two protonated species corresponded to p*K*_a_ = 10 for **1-H**^**+**^ and p*K*_a_ = 9.7 for **1-H**^**+**^_**2**_.

**Scheme 3:**
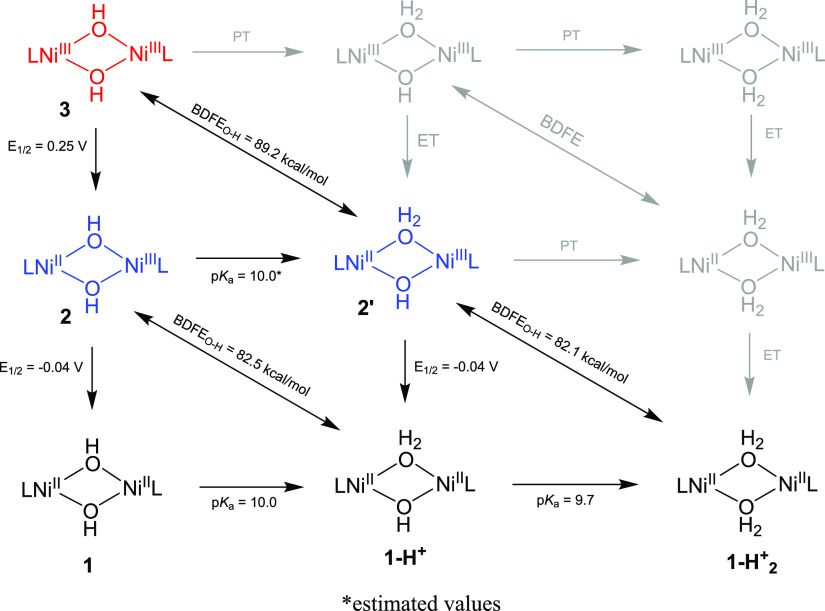
Proposed Square Scheme for the Species Reported in This work Species in gray have not been identified/trapped.

In light of the mechanisms above proposed for the reaction of **3**, **2**, and **2′** with the substrates to form species **2′, 1-H**^**+**^, and **1-H**_**2**_^**+**^, we proposed an overall square scheme (Scheme 3) describing the relationships between all the species reported in this work. The square scheme is a useful tool, which allowed us to correlate all the data about the single components of PCET processes, namely the proton transfer (through p*K*_a_) and electron transfer (through electrochemical potential, *E*_1/2_) in order to have a deeper insight about the overall PCET event. Using methods developed by Bordwell^34^ and later advanced by Mayer for transition metal oxidants,^19^ the BDFE value for each species was estimated using the following formula: BDFE_O-H_ = 1.37 (p*K*_a_) + 23.06 (*E*_1/2_) + *C*_G,DMF_. Our goal was to have BDFE_O-H_, p*K*_a_, and *E*_1/2_ values in hand to understand fully the mechanism and driving forces for oxidation of phenols by **3**, **2**, and **2′**.

We performed cyclic voltammetry on **1** at −40 °C (Figure S51), trying to match closely the reaction conditions. The oxidation potentials for the two quasireversible waves at −40 °C were *E*_1/2_ = −0.04 V and *E*_1/2_ = 0.25 V, assigned to the conversion of **1**-to-**2** and **2**-to-**3**, showing little-to-no differences compared with the reported room-temperature data (*E*_1/2_ = −0.06 V, *E*_1/2_ = 0.24 V).^18^ With these results, a BDFE_O–H_ value for **1-H**^**+**^ of ∼82.5 kcal/mol (p*K*_a_ = 10.0; *E*_1/2_ = −0.04 V) was calculated. For **1-H**^**+**^_**2**_, the potential for the couple **1-H**^**+**^/**2′** appeared to remain unchanged compared to **1** in the presence of 2,6 dimethyl benzoic acid (Figure S52). Therefore, we assumed this potential to be equal to the one measured for the **1/2** couple, resulting in a BDFE_O-H_ estimate for **1-H**^**+**^_**2**_ of ∼82.1 kcal/mol (p*K*_a_ = 9.7; *E*_1/2_ = −0.04 V). The obtained BDFE_O-H_ values, which showed only negligible differences (0.4 kcal/mol), reflected the similar oxidative capability of the species **2** and **2′**.

Similarly, the BDFE_O–H_ of **2′** was estimated. As mentioned above, it was not possible to measure a p*K*_a_ value for the protonation of **2** to give **2′** or indeed the deprotonation of **2′** to yield **2** due to the transient nature, thermal instability, and spectral similarities of both species (see above). Therefore, the p*K*_a_ value for the formation of **2′** was approximated to be the same as the value measured for the monoprotonation of **1** to **1-H**^**+**^. In light of these considerations, the estimated BDFE_O-H_ value for **2′** was ∼89.2 kcal/mol (p*K*_a_ = 10.0; *E*_1/2_ = 0.25 V).

### Discussion

Analysis of the measured *k*_2_ values identified **3** as the kinetically more reactive oxidant among the three, reacting nearly 100-fold faster than **2** and **2′** for all substrates tested (Table 1). The kinetic superiority of **3** with respect to **2** and **2′** is consistent with the calculated BDFE_O–H_ values for **2′**, **1-H**^**+**^, and **1-H**_**2**_^**+**^. The formation of species **2′** is more favored than for **1-H**^**+**^ and **1-H**_**2**_^**+**^ with a BDFE_O–H_ nearly ∼7 kcal/mol higher than the values estimated for **1-H**^**+**^ and **1-H**_**2**_^**+**^. Given the similarities in pKa values for **2′**, **1-H**^**+**^, and **1-H**_**2**_^**+**^, it can be concluded that the enhanced reactivity in **3** is predominantly linked to the presence of two Ni^III^ ions.

Analysis of the kinetic results for **3** (Table 1) showed that the phenol bearing the most electron-rich substituent (i.e., 4-OCH_3_-2,6-DTBP) reacted most rapidly, exhibiting a *k*_2_ value 2 orders of magnitude higher than those of the unsubstituted 2,6-DTBP (*k*_2_ = 520 and 1.16 M^–1^ s^–1^, respectively). Such a behavior, strictly related to a low BDE_O–H_ of 4-OCH_3_-2,6-DTBP (BDE_O–H_ = 78.3 kcal/mol),^35^ was ascribed to a more general trend for all substrates. A linear correlation between the magnitude of the BDE_O–H_ and the *k*_2_ values was observed (Figure S53). Such a linear trend strongly suggested the presence of a PCET event,^20,22,36^ coherent with the identified oxidation products (Table 1).

**Figure 4:**
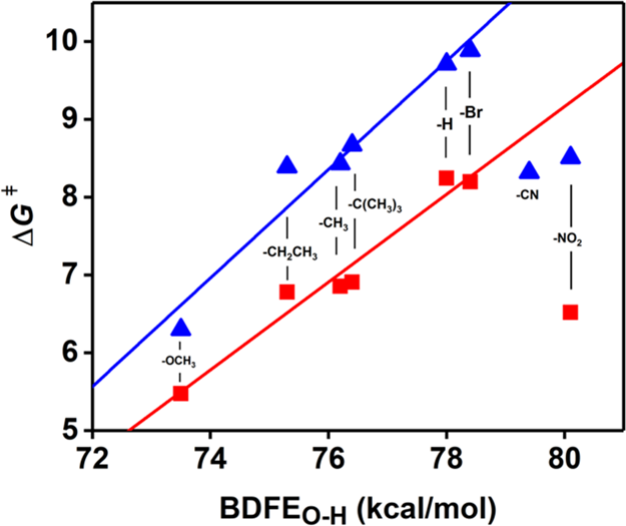
Evans–Polanyi plot (Δ*G*^‡^ vs BDFE_O–H_) for the reaction of **3** (red) and **2** (blue) with a series of 4-X-2,6-DTBP in DMF at −45 °C. Points for X = CN and NO_2_ not included in trend lines.

It has been defined that in a hydrogen atom transfer reaction (HAT, a form of the concerted PCET mechanism, in which protons and electrons move simultaneously from the same donor to the same acceptor), a linear correlation between the Eyring barrier (Δ*G*^‡^) and the Δ*G*° is expected, with a slope close to 0.5, as calculated using Marcus theory.^20,37−40^ Because in an organic HAT reaction, the entropy component of the reaction is almost null (Δ*S*° ≌ 0), Δ*G*° is effectively equal to Δ*H*°(thus BDEs). However, Meyer demonstrated that in transition metal complexes, this assumption (i.e., the negligible nature of the entropic term) is not always correct, and therefore bond dissociation free energies (BDFE), that include both enthalpic and entropic contributions, must be used.^19,20,41^ The Eyring barrier Δ*G*^‡^ for the reaction of **3** with the substrates (calculated from the *k*_2_ values using the Eyring equation)^38−40^ was plotted against the BDFE_O–H_ of the phenols (Figure 4, Table S2, and Figure S54), displaying a good linear correlation. Linear regression calculations gave a slope of 0.56(6) (Table 2). This value was very close to the theoretical value of 0.5 expected for a formal HAT mechanism, suggesting that a HAT mechanism occurred in the phenol oxidation by **3**.

**Table 2:** Correlation Plot Slopes for the Reactivity of **3**, **2**, and **2′**

	Evans–Polanyi plot	Hammett plot	Marcus plot
**3**	0.56(6)	–2.9(3)	–0.21(3)
**2**	0.70(8)	–3.6(5)	–0.27(1)
**2′**	0.58(10)	–2.9(6)	–0.23(2)

The log(^X^*k*_2_/^H^*k*_2_) for **3** was plotted as a function of the Hammett parameter σ^+^_p_ (Figure S55), giving an insight of the electronic effect of the para substituents on the reactivity. Compound **3** displayed a linear dependency of the *k*_2_ values on the nature of the para substituent in 4-X-2,6-DTBP. Linear fits of the plot resulted in negative Hammett slope, ρ = −2.9(3), suggesting that the reaction involved the loss of a negative charge (i.e., the protonation of the negatively charged hydroxide ligand upon the HAT event).^42,43^ This evidence, along with the linear relationship of the *k*_2_ values, were consistent with a HAT mechanism, as previously observed.^44−47^

**Figure 5:**
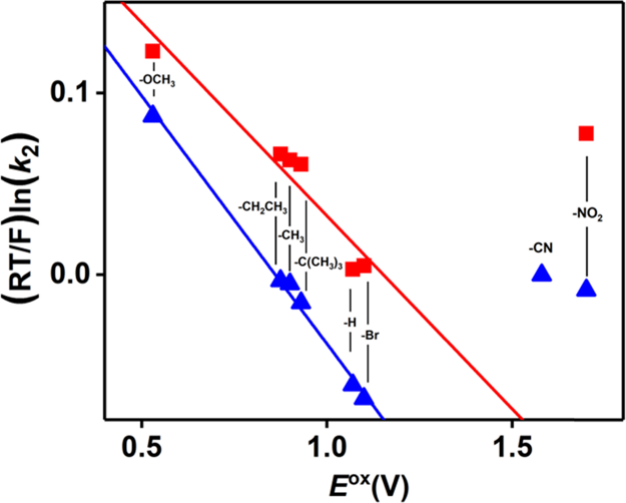
Marcus correlation plot ((RT/F)ln(*k*_2_) vs *E*^ox^) for the reaction of **3** (red) and **2** (blue) with a series of 4-X-2,6-DTBP in DMF at −45 °C. Points for X = CN and NO_2_ not included in trend lines.

Marcus theory has been successfully adapted to understand PCET reactions,^20,37,48,49^ and predicts a linear correlation between the rate constants (as (*RT*/*F*)ln(*k*_2_)) and the one e^–^ oxidation potentials of the phenols. The slope can vary between −1.0 and −0.5 in the presence of non-concerted PCET reactions with proton transfer (PT) and electron transfer (ET) with similar rates. In the case in which the rate-determining step is the proton transfer (and the ET is in equilibrium), a slope of −1.0 is expected. If the electron transfer is the rate-determining step (followed by a fast PT), the slope will be close to −0.5.^36,37,45,50^ In the case of HAT, the slope is instead predicted to be close to 0.0.^26,51,52^ The high-valent species **3** exhibited a linear Marcus plot (Figures 5, S56, except for X = CN and NO_2_), resulting in a slope value of −0.21(3) (Table 2), which is considered indicative of HAT. The experimental evidence and the correlation plots were all in agreement in identifying HAT as the mechanism for the reaction of **3** with the substrates, supporting the overall mechanism proposed in Scheme 3.

Analysis of the *k*_2_ values obtained for the reaction of **2** with the selected substrates showed an overall decrease in their values compared to **3**, suggesting a diminished reactivity caused by the presence of a single Ni^III^ center in the complex, and consistent with the lower BDFE measured for **1-H**^**+**^. The *k*_2_ values showed a linear dependency on the BDE_O–H_ of the phenols (Table 1, Figure S57), with exception of the substrates with X = −CN and −NO_2_. In order to confirm the proposed mechanism, correlation plots such as the Evans–Polanyi plot (Figure 4, S58), the Hammett plot (Figure S59), and the Marcus plot (Figures 5 and S60) were analyzed. All three plots displayed a good linear correlation for all the selected substrates. The calculated slope value of 0.70(8) for the Evans–Polanyi plot appeared to be slightly high compared to the ideal value of 0.5. Nonetheless, we believe that is within the experimental error, and was thus still indicative of a HAT mechanism. The negative slope of the Hammett plot (ρ = −3.6(5)), alongside a value for the Marcus plot slope of −0.27(1) were indications of a HAT mechanism.

Species **2′** has been rationalized as a Ni^II^Ni^III^(μ-OH) (μ-OH_2_) complex (Scheme 2). Consistent with this, the reactivity of **2′** toward the selected 4-X-2,6-DTBP resembled that observed for **2** (Table 1), with similar *k*_2_ values and dependency on the phenol’s BDE_O–H_ (Figure S61). All of the correlation plots for **2′** displayed a good linear correlation, and the analysis of the slopes obtained upon linear regression was consistent with the proposed HAT mechanism, which appears to be the oxidation mechanism of choice for these high-valent systems (Table 2, Figures S62–S64).

The calculated KIE value for **2′**, as well as the already reported values for the reactive species **3**(18) and **2**(18) were all within the classical limit of 7, as widely observed for M–O–X oxidants.^53^ This observation rules out the presence of a tunneling mechanism, as reported for analogues oxo-ligated complexes such as Itoh’s Ni^III^_2_(μ-O)_2_ (KIE = 21.4),^16^ Tolman’s Cu^III^_2_(μ-O)_2_ (KIE = 26–40),^54^ and Que’s Fe^III^Fe^IV^(μ-O)_2_ (KIE = 20).^55^ The measured KIE values are thus consistent with a HAT mechanism and indicate the high *k*_2_ values for **3** are not attained through tunneling, but simply as a result of the high driving force imbued by **2′**.

The reaction outcomes observed for X = −CN and −NO_2_ are not unexpected and have been identified by us and others for comparable high-valent oxidants.^22,56^ The anomalous reactivity could be attributed to a change of mechanism from HAT to a simple proton transfer in which the relatively lower p*K*_a_ of these electron-poor substrates would be the driving force,^57^ or a stepwise PCET oxidation (slow PT followed by fast ET). The former outcome is consistent with the observation of phenolate species in the reaction mixtures for X = −NO_2_.

Finally, the reactivity of **3**, **2**, and **2′** was compared with those of mono- and dinuclear late transition metal complexes, using the oxidation of 2,6-DTBP as a comparative probe (Table 3). **3** showed a *k*_2_ value nearly 10 times higher than most reported Ni oxidants.^26,27,33^**3** reacted almost 100 times faster in the oxidation of 2,6-DTBP than the dinuclear oxo complexes reported,^16,36^ demonstrating its ability as one of the most reactive Ni^III^ complexes reported so far for O–H activation. [Ni^III^(pyalk)_2_]^+^ was the most reactive Ni^III^ species, although a direct comparison is difficult because its rate of reaction was measured at room temperature. **2** and **2′** were considerably less reactive than most of the mononuclear complexes. Overall, achieving the Ni^III^_2_ state has imbued complex **3** with exceptional rates of phenol oxidation. Furthermore, the incorporation of a proton into the high-valent oxidant (as hydroxide) has not impacted negatively its reaction rates with phenolic substrates, providing growing support for the efficacy of such high-valent oxidants.

**Table 3:** Comparison between *k*_2_ Values for the Oxidation of 2,6-DTBP by **2**, **3**, and **2′** and Some Selected Complexes^a^

complex	*k*_2_ (M^–1^ s^–1^)	*T* (°C)	solvent	refs
**3**	1.16(3)	–45	DMF	this work
2	0.045(2)	–45	DMF	this work
2′	0.042(2)	–45	DMF	this work
[Ni^III^(OAc)(pyN_2_^Me2^)]	0.125	–40	acetone	(27)
[Ni^III^(ONO_2_)(pyN_2_^Me2^)]	1.96	–40	acetone	(27)
[(L_1_^H^)Ni^III^(μ-O)_2_Ni^III^(L_1_^H^)]^2+^	0.0166	–50	acetone	(16)
[(MeAN)Cu^III^(μ-O)_2_Ni^III^L]^+^	0.03	–50	CH_2_Cl_2_	(36)
[Ni^III^(pyalk)_2_]^+^	189.3	25	CH_2_Cl_2_	(59)

a pyN_2_^Me_2_^ = *N*,*N*′-bis(2,6-dimethylphenyl)2,6-pyridinedicarboxamidate; L_1_^H^ = *N*,*N*′-bis[2-(2-pyridyl)ethyl]-2-phenylethylamine; MeAN = *N*,*N*,*N*′,*N*′,*N*′-pentamethyl-dipropylenetriamine; L = [HC(CMeNC_6_H_3_(iPr)_2_)_2_]; ^s^PhINTs = *N*-(*p*-toluenesulfonyl)imino(2-*tert*-butylsulfonyl)phenyliodinane; and pyalk = 2-pyridinyl-2-propanoate.

## Conclusions

In conclusion, we explored the reactivity of bis-μ-hydroxide-Ni_2_ complexes **3** and **2** toward a large family of phenolic O–H bonds. Upon reaction of **3** with phenols, we observed the formation of a new transient species, **2′**, which has been demonstrated to react with substrates in a very similar fashion to **2**. The obtained kinetic data for the three oxidants indicated that all three species performed the oxidation through a HAT mechanism. Bis-μ-hydroxide-Ni^III^_2_**3** displayed *k*_2_ values higher than either mixed-valent **2** and **2′** and, critically, that any Ni^III^_2_(μ-O)_2_ complex reported to date. These results clearly demonstrated that high-valent hydroxide-bridged dinuclear complexes are capable oxidants and match the kinetic reactivity of the most reactive comparable oxo-bridged entities, giving new cues into the current understanding of the activity of several dinuclear metalloenzymes.

## Supporting Information Available

All experimental and synthetic methods; kinetic data for the reactions of **2**, **2′**, and **3** with the substrates; ^1^H NMR of postreaction mixtures; titration of **1** with acids; cyclic voltammetry; and correlation plots (PDF)
