# Supplementary material for: Reactivity Properties of Mixed- and High-Valent Bis(μ-Hydroxide)-Dinickel Complexes
Source: ACS Omega. 2021 Oct 15;6(42):28162–70. doi: 10.1021/acsomega.1c04225 (PMC8554787; doi:10.1021/acsomega.1c04225)
Supplement: Supplementary file 1 — ao1c04225_si_001.pdf [file ao1c04225_si_001.pdf]

Supporting Information for

Reactivity Properties of Mixed- and High-Valent Bis(μ-Hydroxide)-Dinickel Complexes

Giuseppe Spedalotto,[a] Marta Lovisari,[a] Aidan R. McDonald[*a]

[a] School of Chemistry, Trinity College Dublin, The University of Dublin, College Green, Dublin 2 (Ireland)

E-mail: aidan.mcdonald@tcd.ie

General Information

Materials

All reactions were performed under atmospheric conditions. Reagents and solvents were purchased from commercial suppliers and have been used as received, unless otherwise stated.

Physical Methods

Electronic absorption spectra were recorded on a Agilent 8453 diode array spectrophotometer (190-1100 nm range) equipped with a cryostat unit from Unisoku Scientific Instruments (Osaka, Japan). ^{1}H nuclear magnetic resonance (NMR) analyses were performed on an Agilent MR 400 MHz (400.13 MHz for ^{1}H-NMR). Electrospray ionisation mass spectrometry (ESI-MS) analyses were performed using a Micromass Time of Flight (ToF), interfaced with a Waters 2690 HPLC. Electron paramagnetic resonance (EPR) spectra of frozen solutions were acquired on a Bruker EMX X-band EPR, equipped with an Oxford Instruments CE 5396, ESR9 Continuous Flow Cryostat, a precision Temperature Controller and an Oxford Instruments TTL20.0/13 Transfer Tube. EPR samples were prepared by freezing the EPR tubes containing the analyte solutions, previously prepared at the cryostat as listed below, in liquid nitrogen. Cyclic voltammetry (CV) experiments have been conducted with a CH Instrument 600E electrochemical analyser, using a GC working electrode, a Pt wire counter electrode, and an Ag/$AgNO_3$ 0.01 M reference electrode.

Kinetic experiments

Different amounts of substrate, as a DMF solution, were added to a 0.2 mM solution in N,N dimethylformamide (DMF) of **2** or **3** at -45 °C (prepared in situ according to the reported procedure)[1] causing the immediate decay of the spectral features of the oxidant species. For species **2**, the monitored wavelength was λ_{max} = 560 nm, while in the case of species **3** was monitored λ_{max} = 600 nm. The decay of these features upon addition of substrates was fitted using a *pseudo*-first order kinetic model, obtaining a k_{obs} for each reaction. For complex **2**, the decay pattern was composed of single exponential curve. In the case of the reaction of **3** with the substrates, a biphasic decay pattern was observed. The first exponential decay, corresponding to the reaction between **3** and the substrate to give **2'** and the 1e$^-$ oxidised substrate, was considered for the kinetic studies relative to **3**. The second phase, relative to the reaction of **2'** with excess substrates, was instead considered for the kinetic studies of **2'**. Reporting the k_{obs} values obtained against the concentrations of the correspondent substrate allowed the determination of the *second order* rate constant (k_2) for the examined substrate as the slope of the resulting k_{obs} vs. [substrate] plot.

Preparation of complexes 2 and 3

The complexes **2** and **3** were prepared according to the procedure reported in our previous work [1]

Preparation of 4-NO_2-2,6-di-*tert*-butyl-phenol

The 4-NO_2-2,6-di-*tert*-butyl-phenol was synthesised according the reported procedure.[2]

Preparation of 4-X-2,6-di-tert-butyl phenoxy radicals (X = –OCH_3, –$C(CH_3)_3$)

The 4-X-2,6-di-tert-butyl phenoxy radicals (X = –OCH_3, –$C(CH_3)_3$) were synthesised according to reported procedures.[3, 4]

NMR experiments in DMF-D_7

Compound ^{Me_2}DMMA**H_2**

δ ^{1}*H* (400 MHz, [D_7]-DMF): 1.78 (6H, s, -C*H*3), 2.24 (12H, s, Ar-C*H*3), 7.09 (6H, m, Ar-C*H*), 9.14 (2H, s,-N*H*).

Compound **1**

δ ^{1}*H* (400 MHz, [D_7]-DMF): -9.22 (2H, s, -*OH*), 2.08 (12H, s, -C*H3*), 2.36 (24H, s, Ar-C*H3*), 6.60 (12H, m, Ar-C*H*).

4-OCH_3-2,6-di-*tert*-butyl-phenol

δ ^{1}*H* (400 MHz, [D_7]-DMF): 1.43 (18H, s, -C(C*H3*)3), 3.77 (3H, s, -OC*H3*), 6.57 (1H, s, -O*H*), 6.74 (2H, s, Ar-C*H*)

2,6-di-tert-butyl-1,4-benzoquinone

δ ^{1}*H* (400 MHz, [D_7]-DMF): 1.29 (18H, s, -C(C*H3*)3), 6.58 (2H, s, Ar-C*H*)

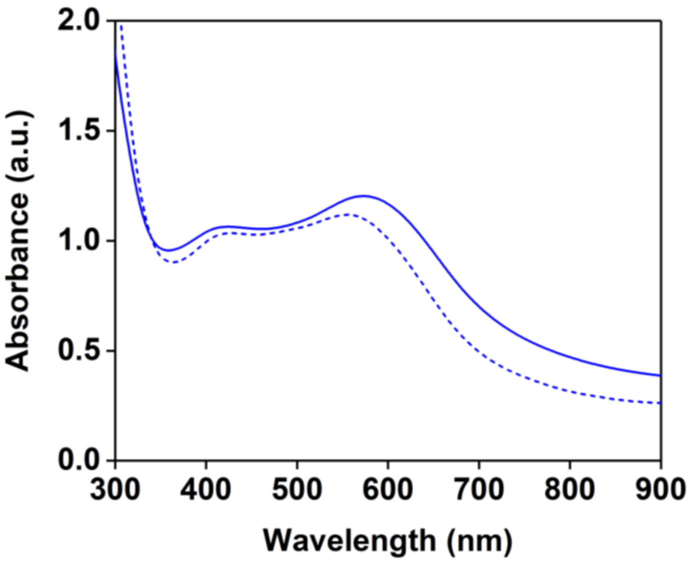

Figure S1 – Comparison between the electronic absorption spectra of **2’** (blue dashed line) and **2** (blue solid line), in DMF at -45 °C.

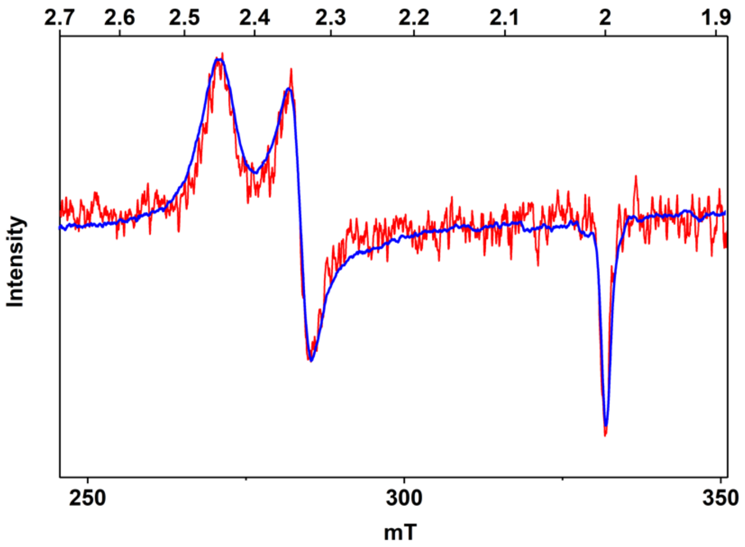

Figure S2 – X-band EPR of **2’** (red trace) and comparison with **2** (blue trace).[1] X-band EPR spectrum was acquired from a frozen DMF solution, measured at 77 K, 2.01 mW microwave power, with 0.3 mT modulation amplitude.

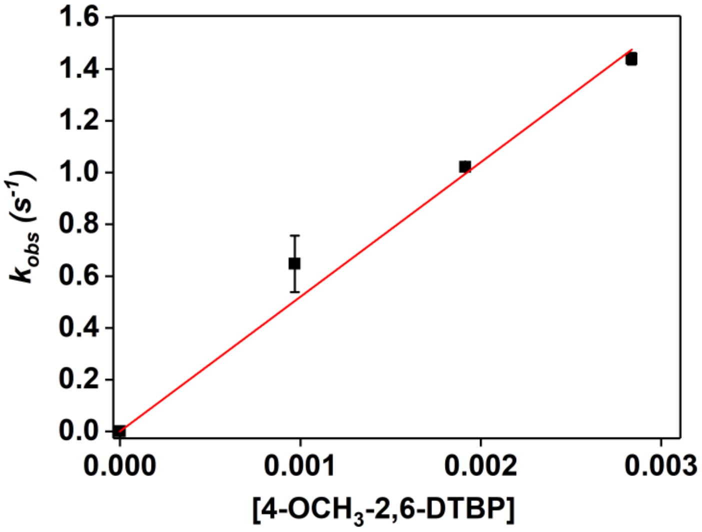

Figure S3 - Plot of k_{obs} vs. [4-OCH$_3$-2,6-DTBP], for the reaction between **3** and 4-OCH$_3$-2,6-DTBP at -45 °C in DMF. k_2 = 520(11) $M^{-1}s^{-1}$.

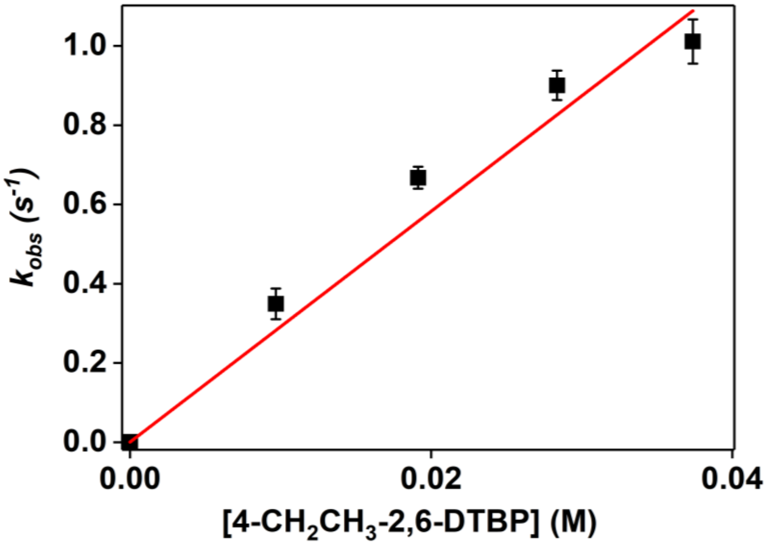

Figure S4 - Plot of k_{obs} vs. [4-CH$_2$CH$_3$-2,6-DTBP], for the reaction between **3** and 4-CH$_2$CH$_3$-2,6-DTBP at -45 °C in DMF. k_2 = 29(1) $M^{-1}s^{-1}$.

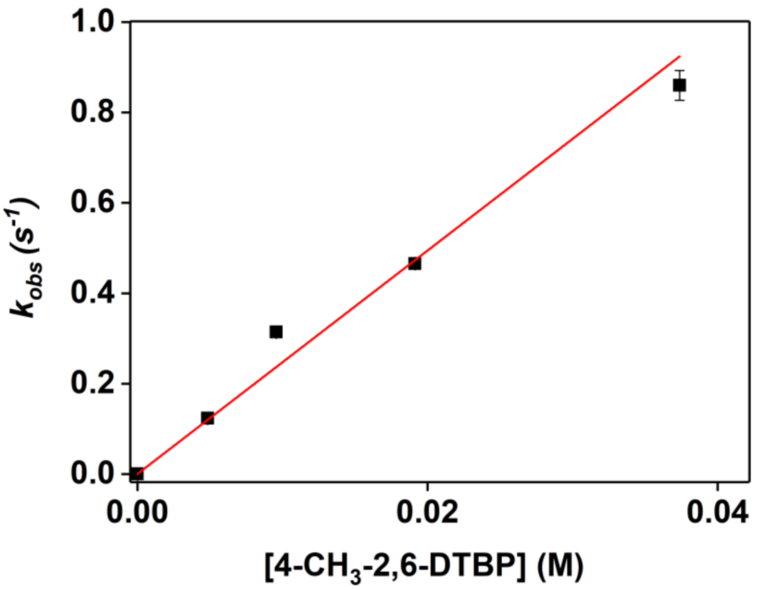

Figure S5 - Plot of k_{obs} vs. [4-CH_3-2,6-DTBP], for the reaction between **3** and 4-CH_3-2,6-DTBP at -45 °C in DMF. k_2 = 24.7(11) $M^{-1}s^{-1}$.

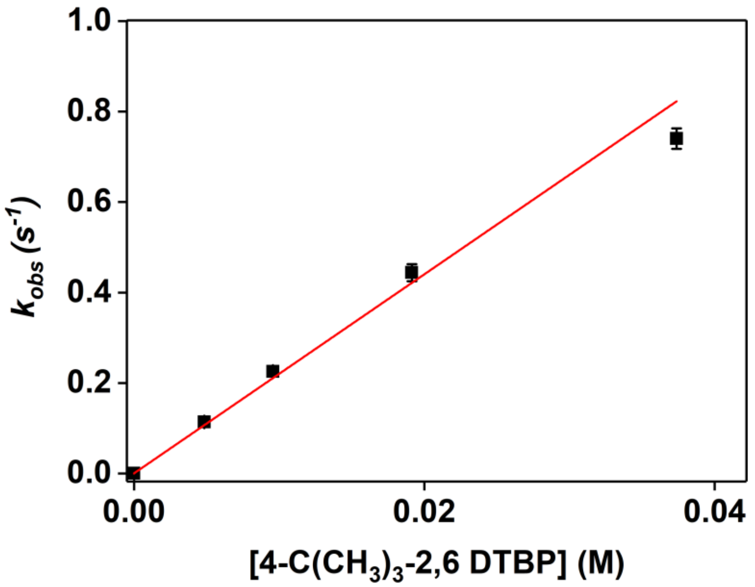

Figure S6 - Plot of k_{obs} vs. [4-$C(CH_3)_3$-2,6-DTBP], for the reaction between **3** and 4- $C(CH_3)_3$-2,6-DTBP at -45 °C in DMF. k_2 = 22(1) $M^{-1}s^{-1}$.

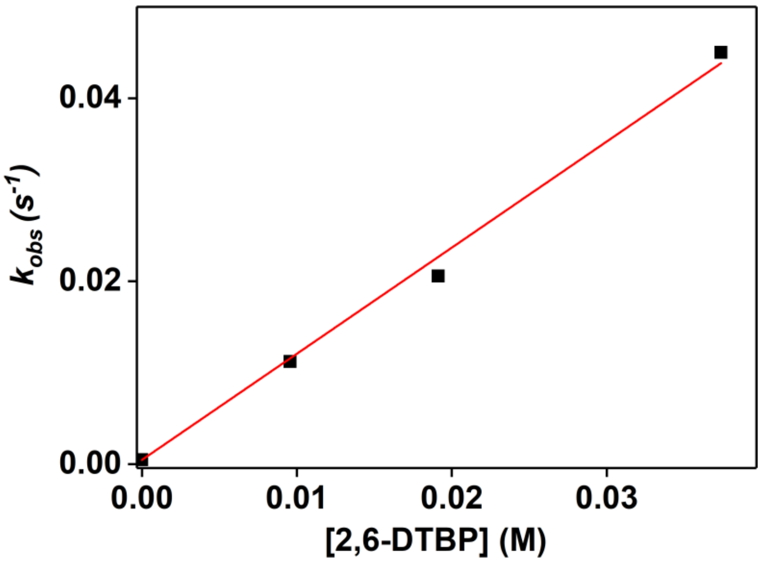

Figure S7 - Plot of k_{obs} vs. [2,6-DTBP], for the reaction between **3** and 2,6-DTBP at -45 °C in DMF. k_2 = 1.16(3) $M^{-1}s^{-1}$.

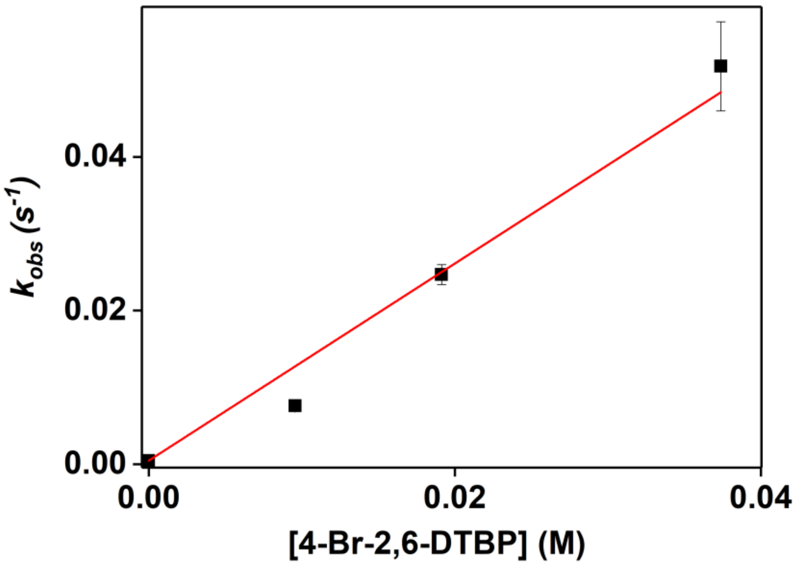

Figure S8 - Plot of k_{obs} vs. [4-Br-2,6-DTBP], for the reaction between **3** and 4-Br-2,6-DTBP at -45 °C in DMF. k_2 = 1.28(4) $M^{-1}s^{-1}$.

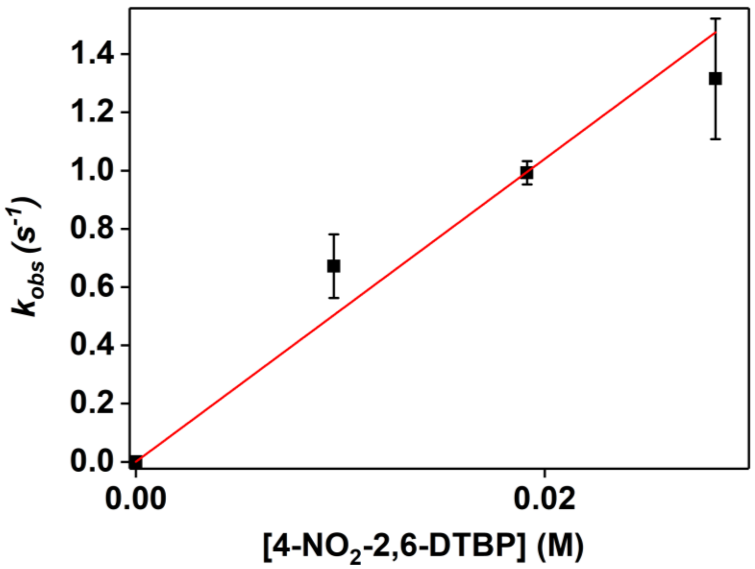

Figure S9 - Plot of k_{obs} vs. [4-NO$_2$-2,6-DTBP], for the reaction between **3** and 4-NO$_2$-2,6-DTBP at -45 °C in DMF. k_2 = 52(24) $M^{-1}s^{-1}$.

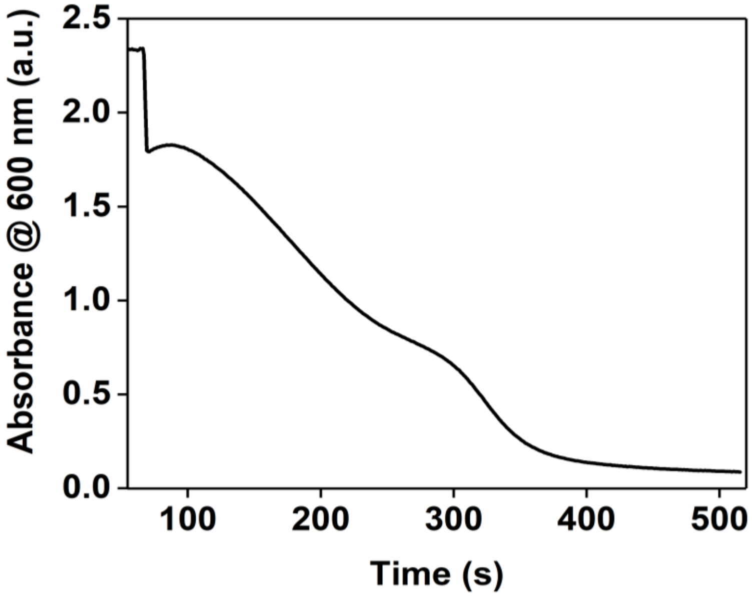

Figure S10 – Variation of the absorbance at λ = 600 nm over the reaction time for the reaction between **3** and 4-CN-2,6-di-*tert*-butyl phenol in DMF at -45 °C.

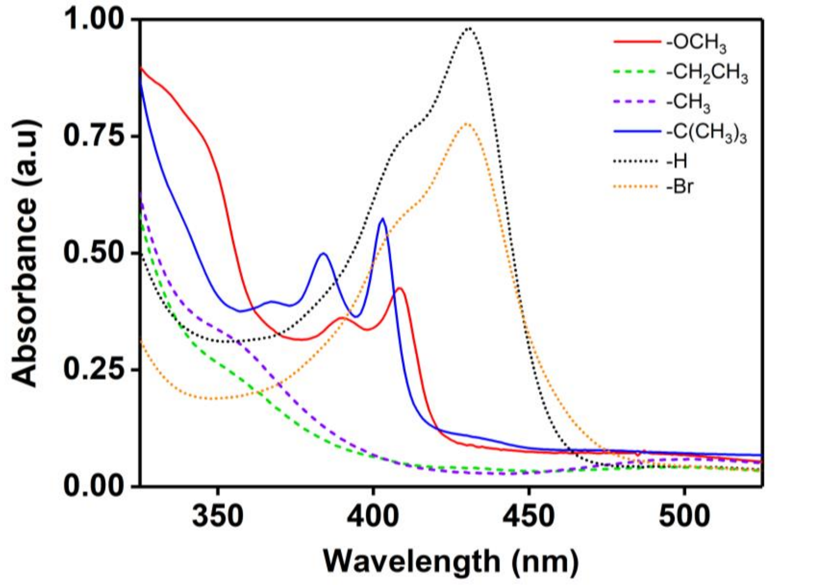

Figure S11 - End reaction products for the reaction between **3** and 4-X-2,6-DTBP (X = $–OCH_3$, $–CH_3CH_2$, $–CH_3$, $–C(CH_3)_3$, –H, –Br).

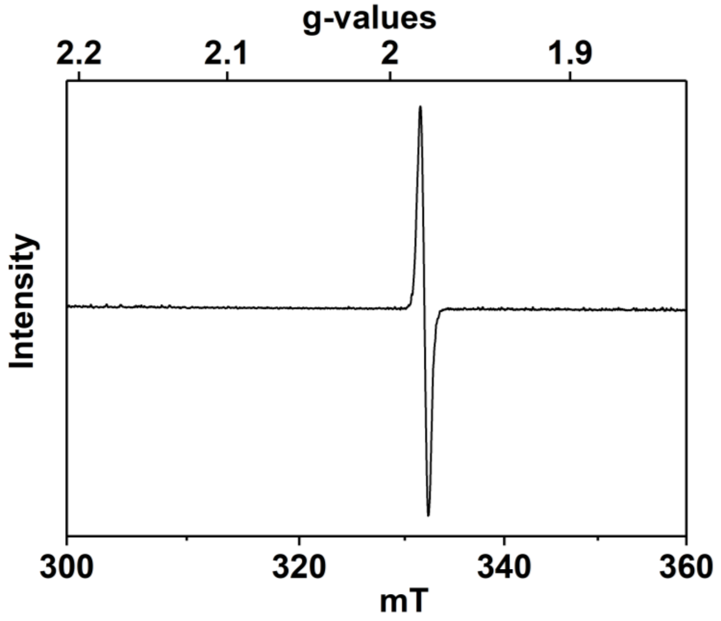

Figure S12 – X-band EPR spectrum of the post reaction mixture for the reaction between **3** and 4-OCH_3-2,6-DTBP at -45 °C in DMF, measured at measured at 77 K, 0.2 mW microwave power, with 0.2 mT modulation amplitude.

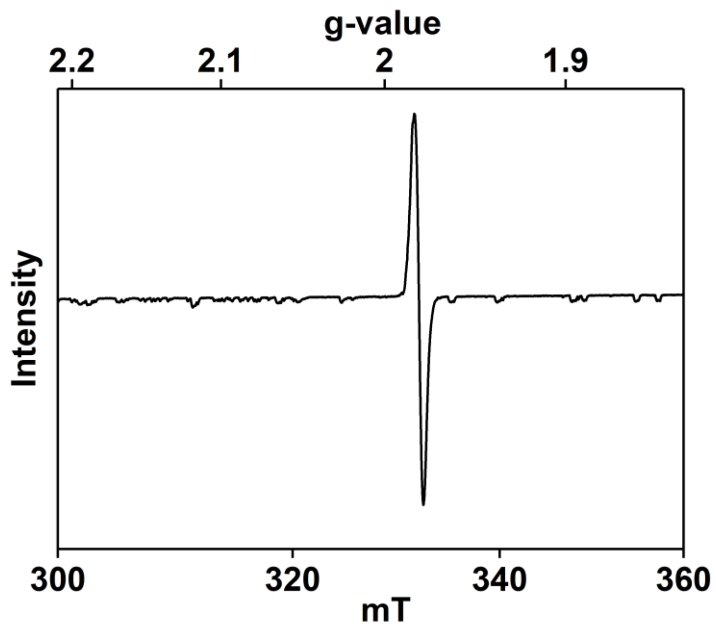

Figure S13 - X-band electron paramagnetic resonance spectrum of the post reaction mixture for the reaction between **3** and 4-$C(CH_3)_3$-2,6-DTBP at -45 °C in DMF, measured at 77 K, 0.2 mW microwave power, with 0.2 mT modulation amplitude.

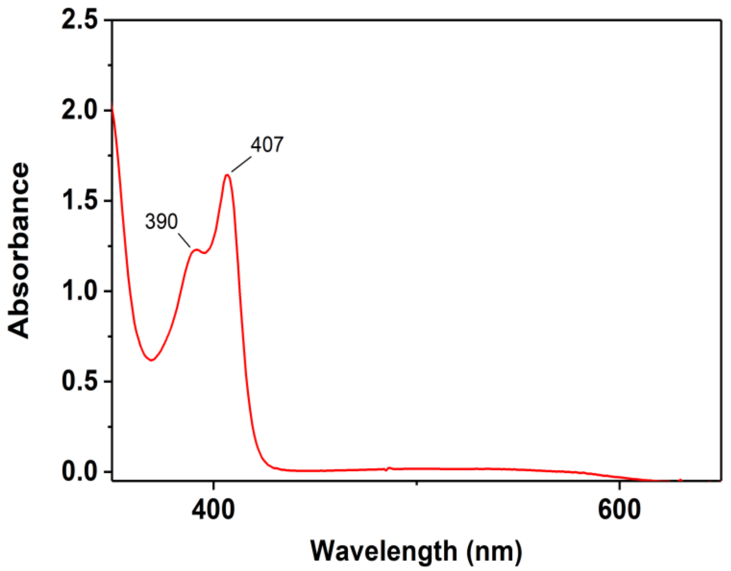

Figure S14 - UV-Vis spectrum of independently synthesised 4-OCH_3-2,6-di-*tert*-butyl phenoxy radical in DMF at 25 °C (ε_{407nm} = 2090 ± 100 $M^{-1}cm^{-1}$).

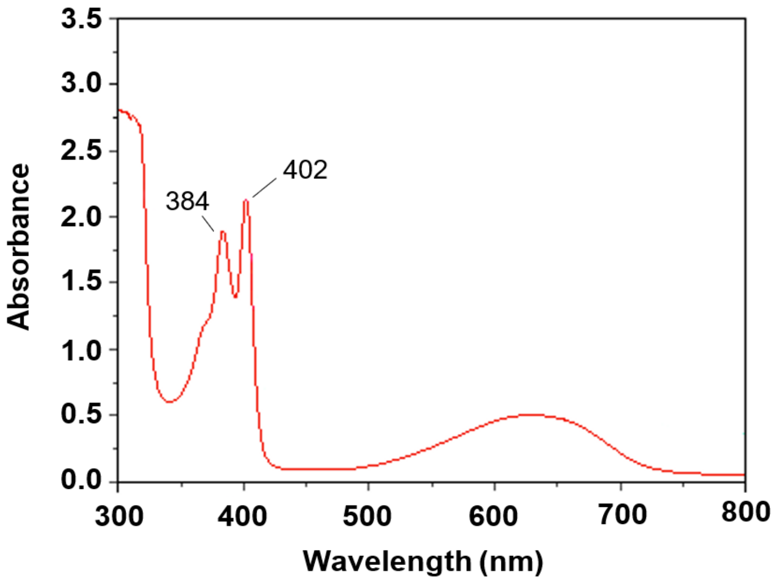

Figure S15 - UV-Vis spectrum of independently synthesised 2,4,6-tris-*tert*-butyl phenoxy radical in DMF at 25 °C (ε_{407nm} 1830 ± 200 $M^{-1}cm^{-1}$).

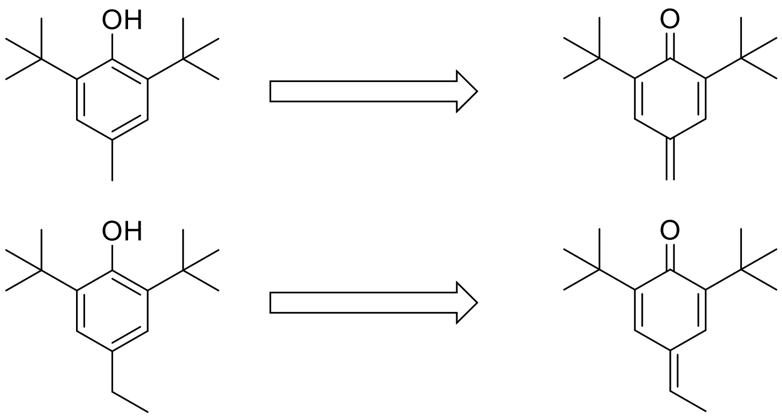

Figure S16 - Identified products from the oxidation of 4-CH_3-2,6-DTBP and 4-CH_2CH_3-2,6-DTBP by **3**. GC-MS and ESI-MS were used for this analysis.

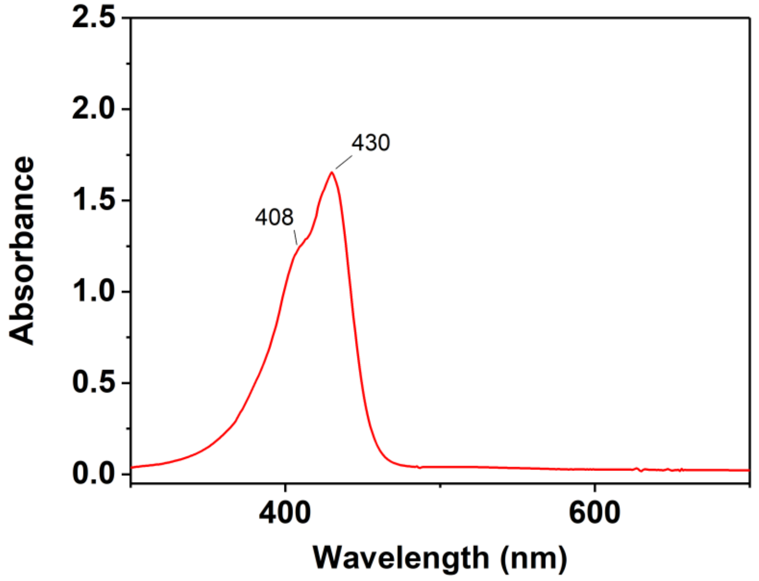

Figure S17 - UV-Vis spectrum of independently synthesised 3,3',5,5'-tetra*tert*-butyl-4,4'-diphenoquinone (DPQ) in DMF at -45 °C (ε_{430nm} = 32380 ± 500 $M^{-1}cm^{-1}$).

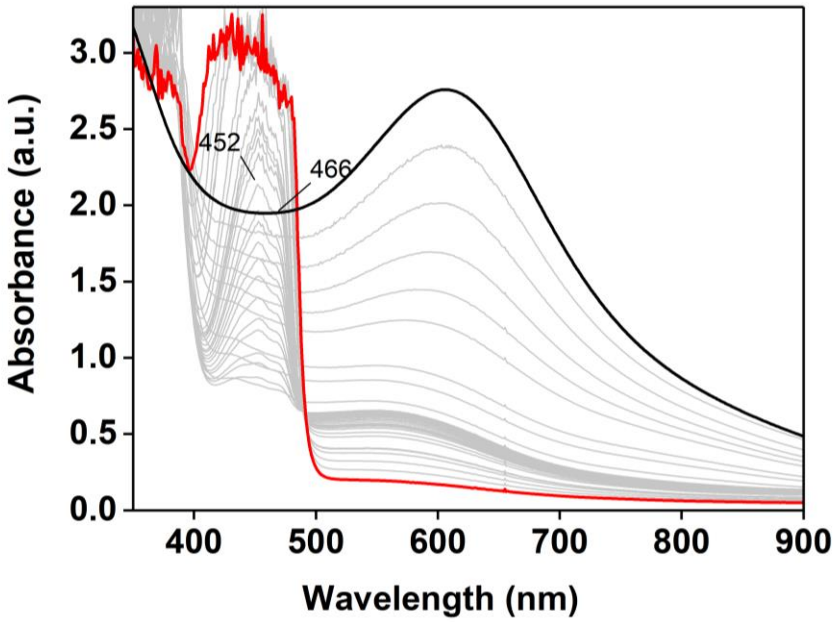

Figure S18 - Electronic absorption spectra showing the formation of the 4-NO_2-2,6-DTB phenolate upon reaction of 4-NO_2-2,6 DTBP with **3** at -45 °C in DMF.

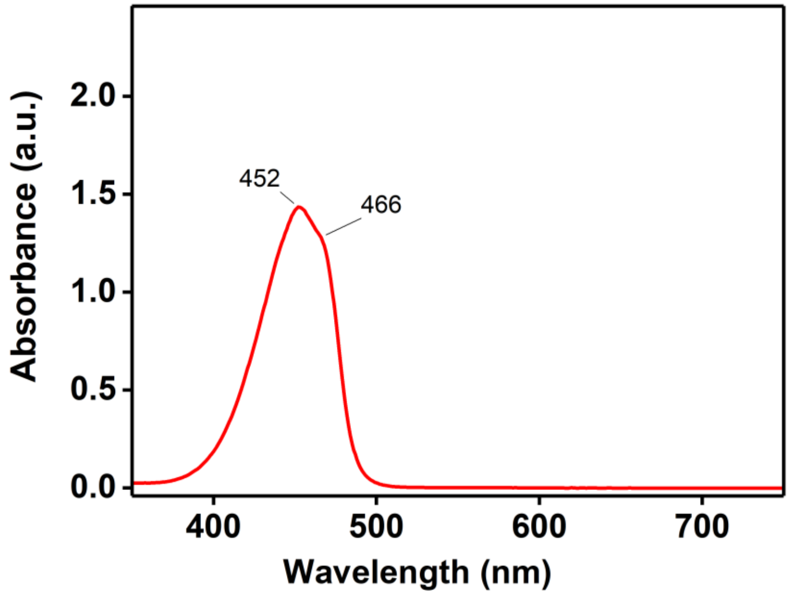

Figure S19 - UV-Vis spectrum of independently synthesised 4-NO_2-2,6-di-*tert*-butyl phenoxide ion in DMF at 25 °C

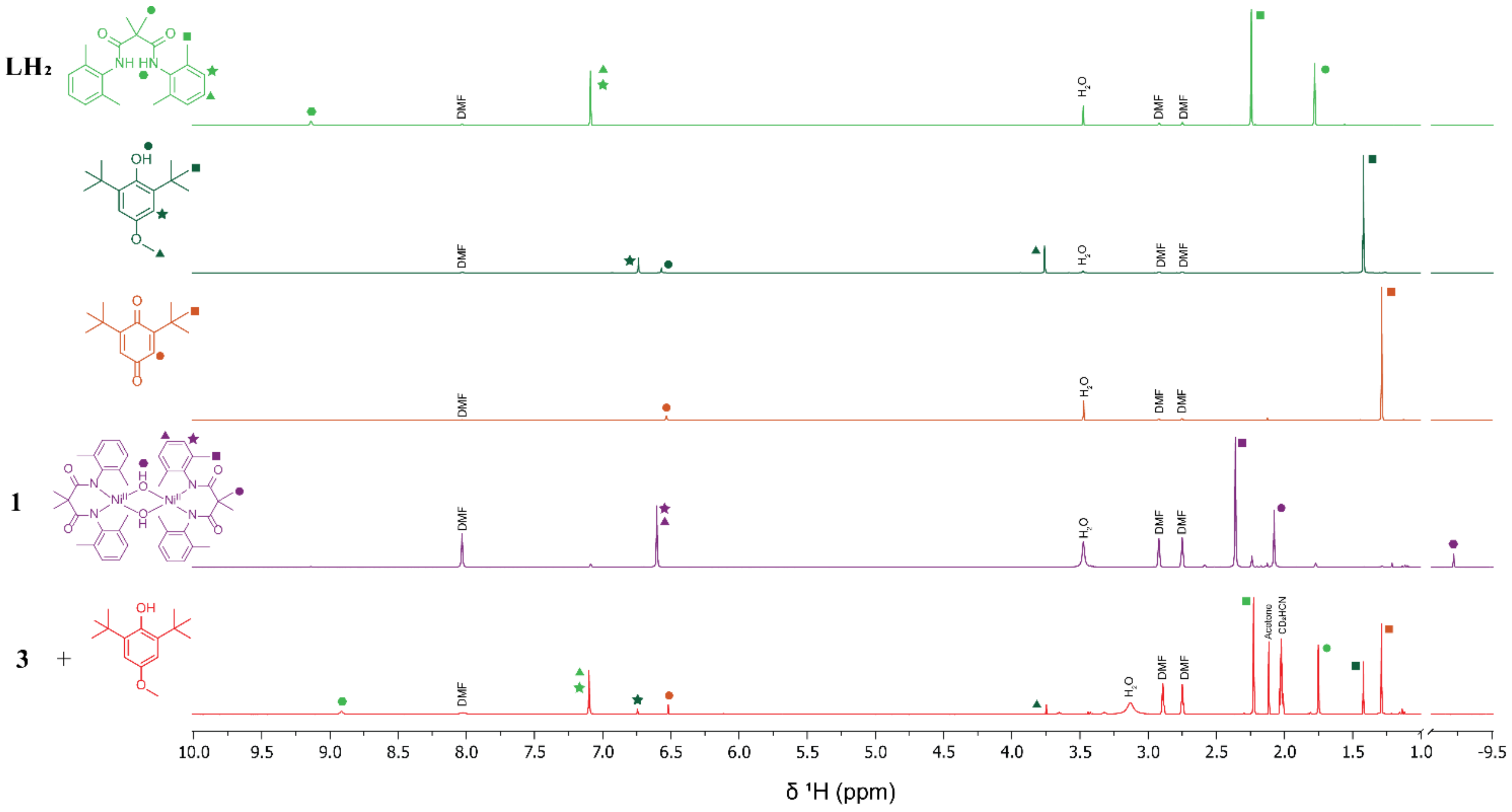

Figure S20 – Room temperature end reaction mixture ^{1}H-NMR for the reaction of **3** with 4-OCH_3-2,6-DTBP in DMF-D_7 at – 45 °C.

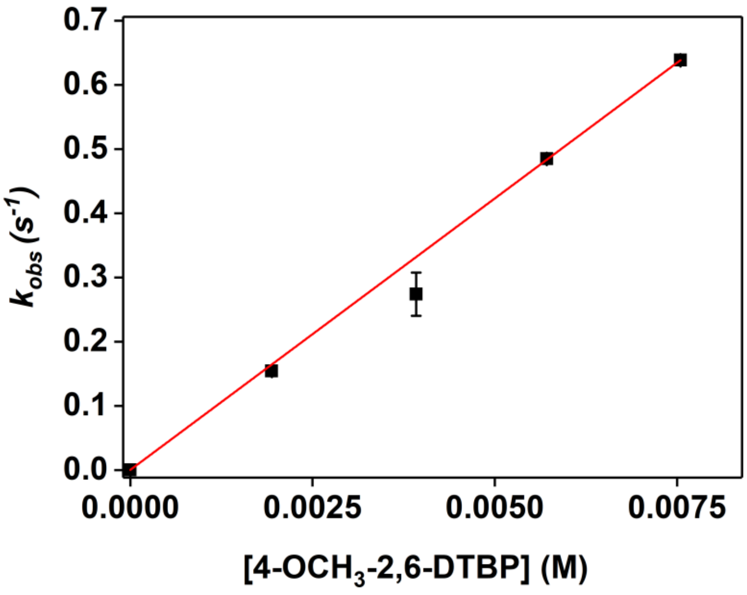

Figure S21 - Plot of k_{obs} vs. [4-OCH_3-2,6-DTBP], for the reaction between **2** and 4-OCH_3-2,6-DTBP at -45 °C in DMF. k_2 = 84.6(3) $M^{-1}s^{-1}$.

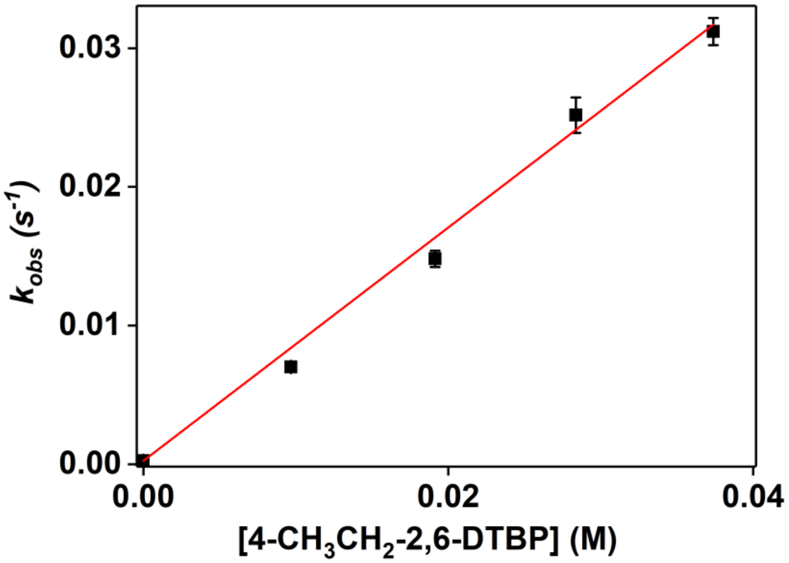

Figure S22 - Plot of k_{obs} vs. [4-CH_2CH_3-2,6-DTBP], for the reaction between **2** and 4-CH_2CH_3-2,6-DTBP at -45 °C in DMF. k_2 = 0.84(2) $M^{-1}s^{-1}$.

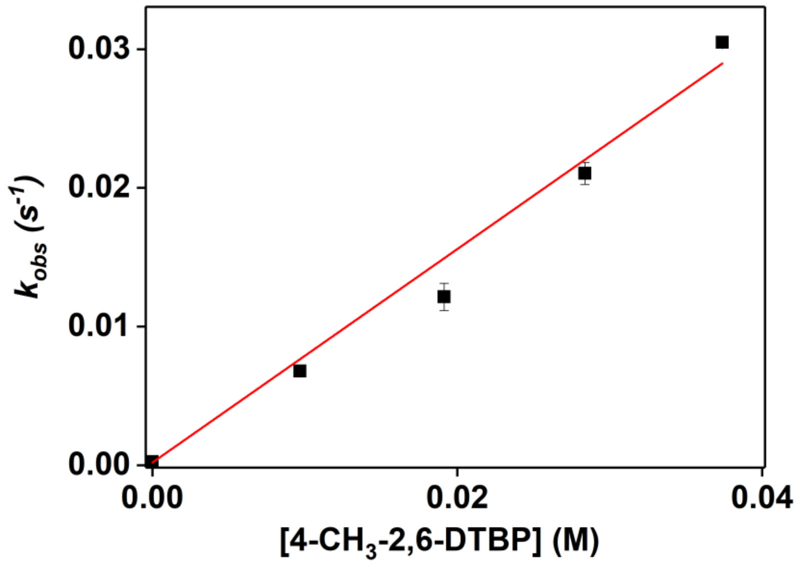

Figure S23 - Plot of k_{obs} vs. [4-CH_3-2,6-DTBP], for the reaction between **2** and 4- CH_3-2,6-DTBP at -45 °C in DMF. k_2 = 0.76(4) $M^{-1}s^{-1}$.

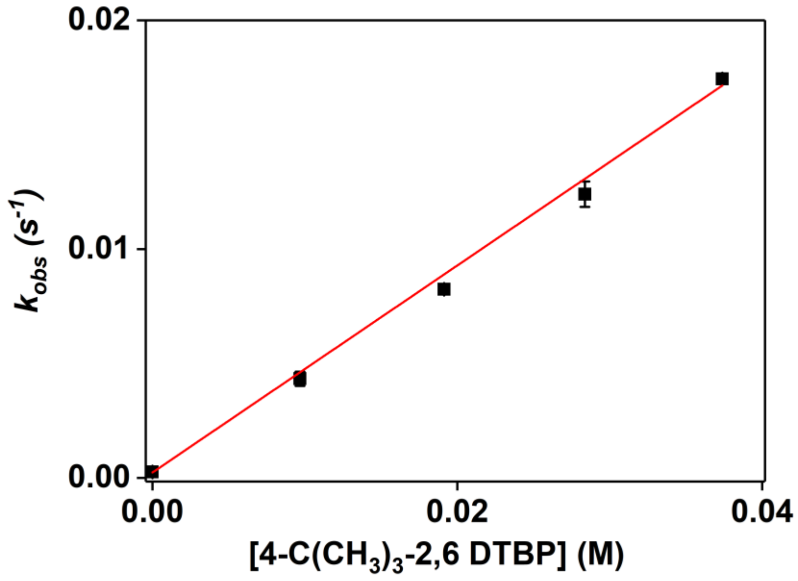

Figure S24 - Plot of kobs vs. [4-C(CH_3)$_3$-2,6 DTBP], for the reaction between **2** and 4-C(CH_3)$_3$-2,6 DTBP at -45 °C in DMF. k_2 = 0.45(1) $M^{-1}s^{-1}$.

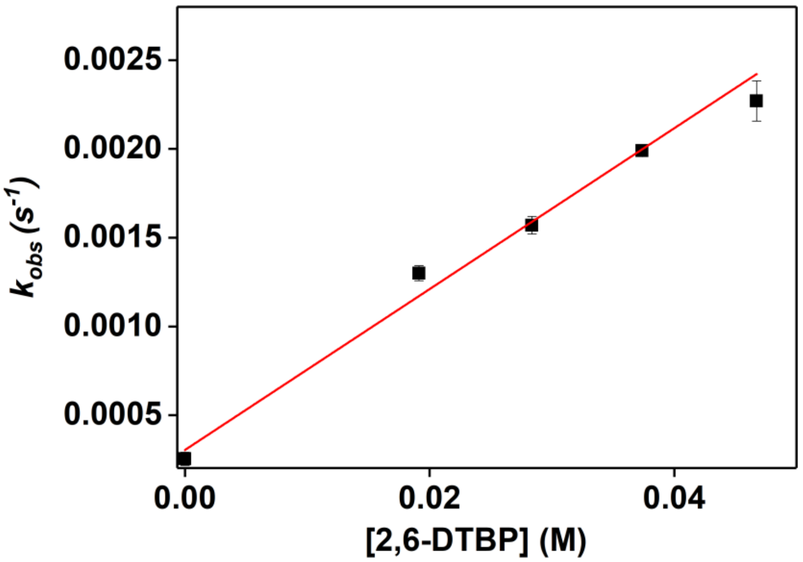

Figure S25 - Plot of k_{obs} vs. [2,6-DTBP], for the reaction between **2** and 2,6-DTBP at -45 °C in DMF. k_2 = 0.045(2) $M^{-1}s^{-1}$.

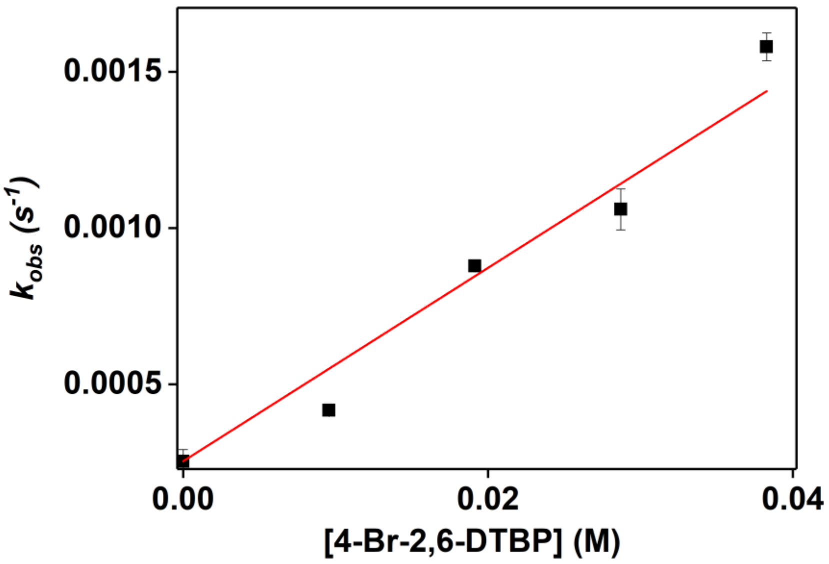

Figure S26 - Plot of k_{obs} vs. [4-Br-2,6-DTBP], for the reaction between **2** and 4-Br-2,6-DTBP at -45 °C in DMF. k_2 = 0.031(3) $M^{-1}s^{-1}$.

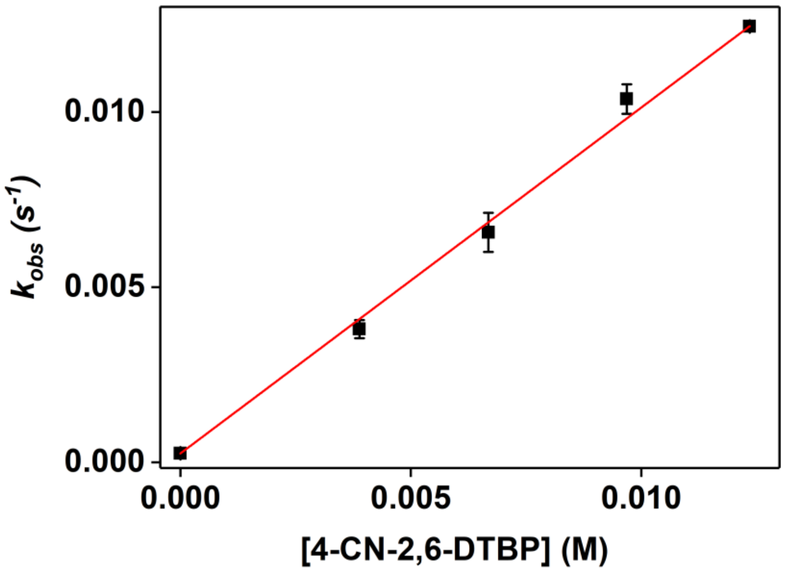

Figure S27 - Plot of k_{obs} vs. [4-CN-2,6-DTBP], for the reaction between **2** and 4-CN-2,6-DTBP at -45 °C in DMF. k_2 = 0.99(1) $M^{-1}s^{-1}$.

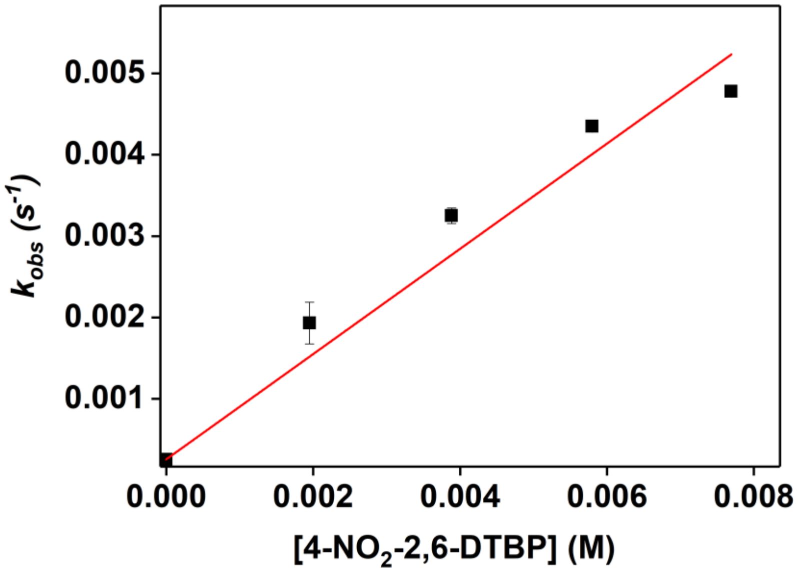

Figure S28 - Plot of k_{obs} vs. [4-NO_2-2,6-DTBP], for the reaction between **2** and 4-NO_2-2,6-DTBP at -45 °C in DMF. k_2 = 0.65(3) $M^{-1}s^{-1}$.

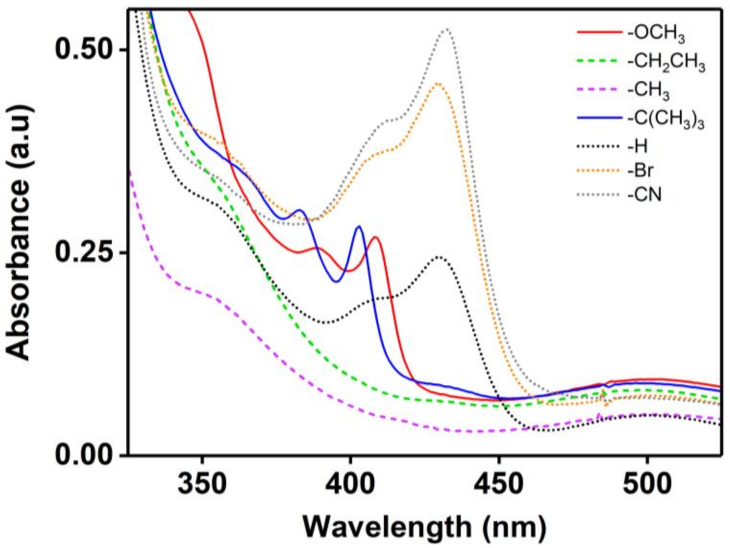

Figure S29 - End reaction products for the reaction between **2** and 4-X-2,6-DTBP (X = –OCH3, –CH3CH2, –CH3, –C(CH3)3, –H, –Br, –CN).

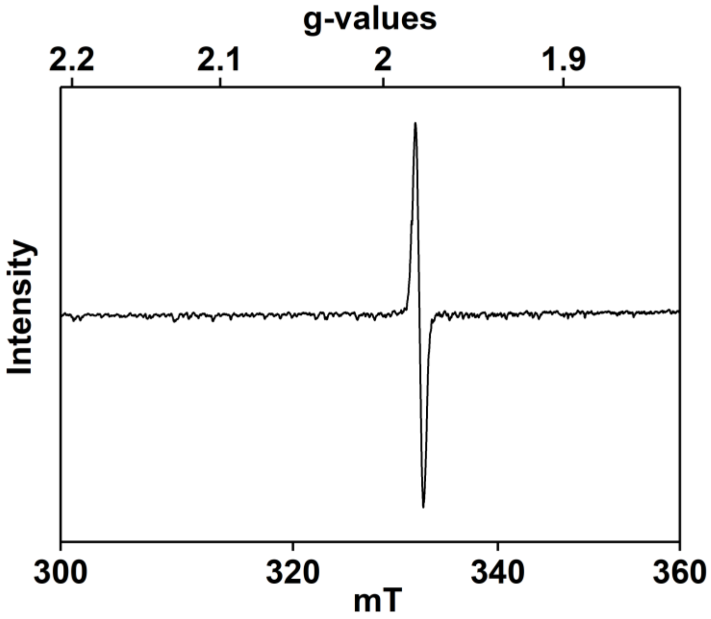

Figure S30 - X-band electron paramagnetic resonance spectrum of the post reaction mixture for the reaction between **2** and 4-OCH3-2,6-DTBP at -45 °C in DMF, measured at measured at 77 K, 0.2 mW microwave power, with 0.2 mT modulation amplitude.

.

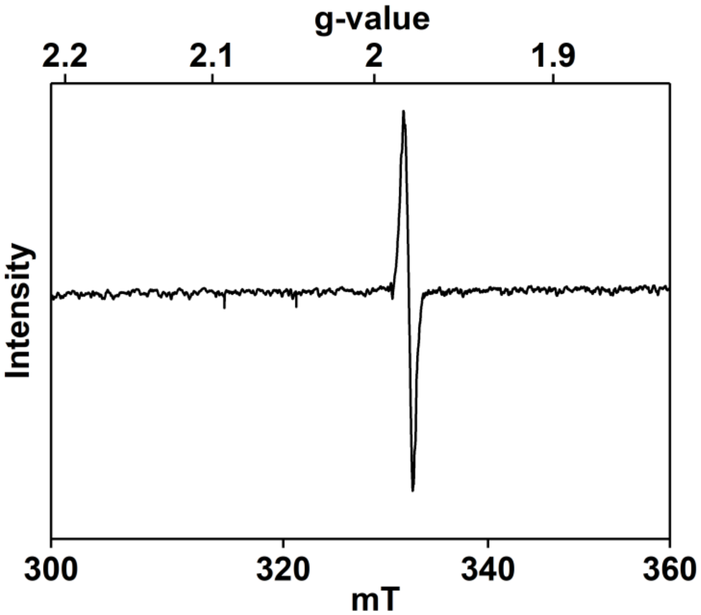

Figure S31 - X-band electron paramagnetic resonance spectrum of the post reaction mixture for the reaction between **2** and 4-C(CH_3)$_3$-2,6-DTBP at -45 °C in DMF, measured at measured at 77 K, 0.2 mW microwave power, with 0.2 mT modulation amplitude.

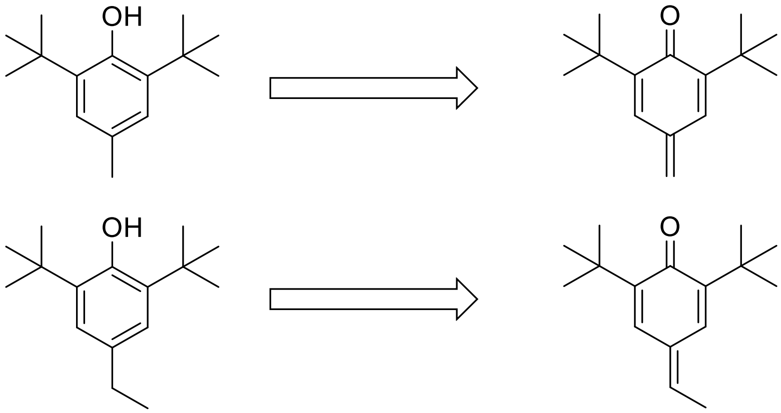

Figure S32 - Identified products from the oxidation of 4-CH_3-2,6-DTBP and 4-CH_2CH_3-2,6-DTBP by **2**. GC-MS and ESI-MS were used for this analysis.

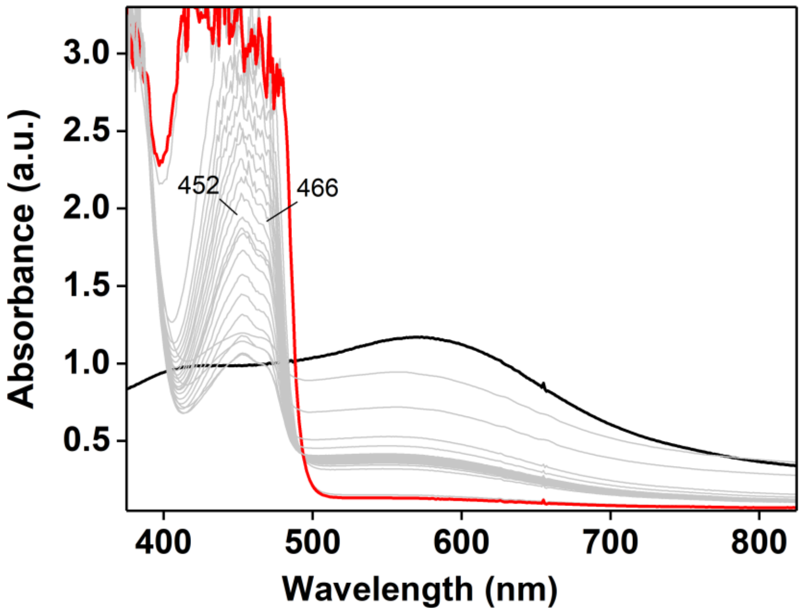

Figure S33 - Electronic absorption spectra showing the formation of the 4-NO_2-2,6 DTB phenolate upon reaction of 4-NO_2-2,6 DTBP with **2** at -45 °C in DMF.

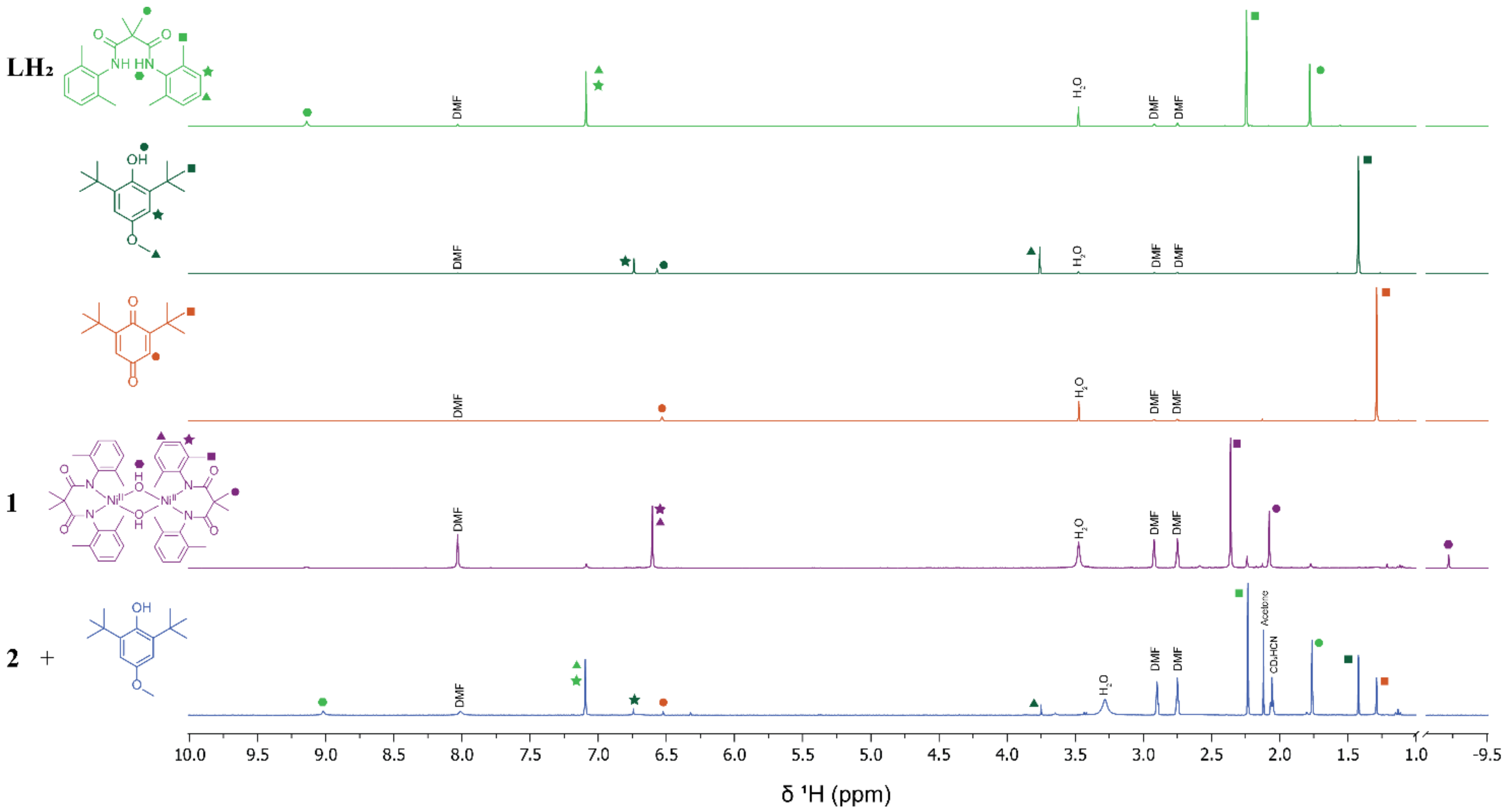

Figure S34 – Room temperature end reaction mixture ^{1}H-NMR for the reaction of **2** with 4-OCH$_3$-2,6-DTBP in DMF-D$_7$ at – 45 °C.

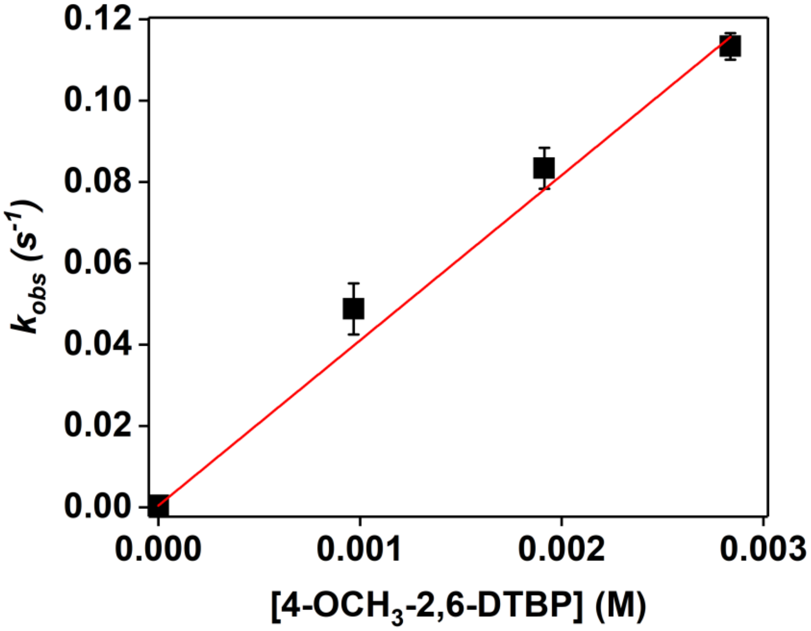

Figure S35 - Plot of k_{obs} vs. [4-OCH_3-2,6-DTBP], for the reaction between **2’** and 4-OCH_3-2,6-DTBP at -45 °C in DMF. k_2 = 41(1) $M^{-1}s^{-1}$.

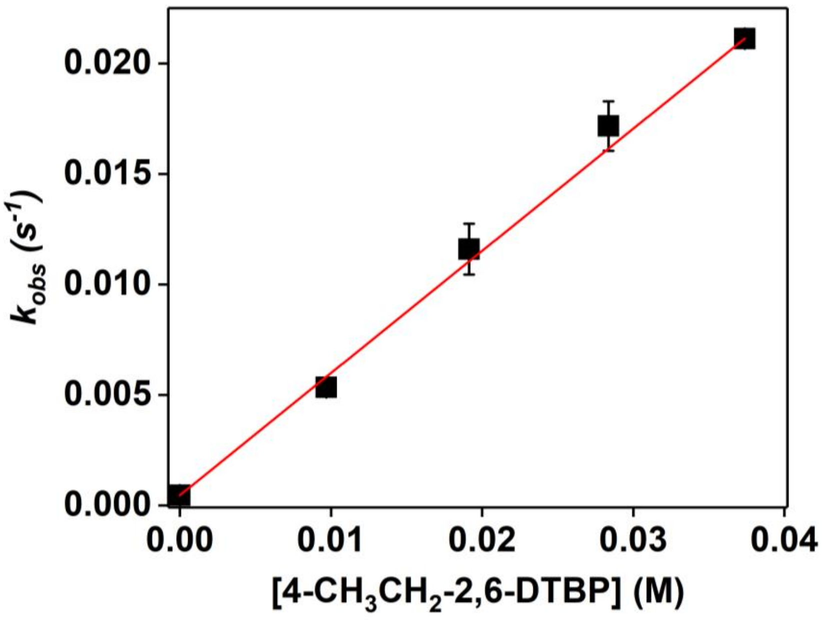

Figure S36 - Plot of k_{obs} vs. [4-CH_3CH_2-2,6-DTBP], for the reaction between **2’** and 4-CH_3CH_2-2,6-DTBP at -45 °C in DMF. k_2 = 0.553(3) $M^{-1}s^{-1}$.

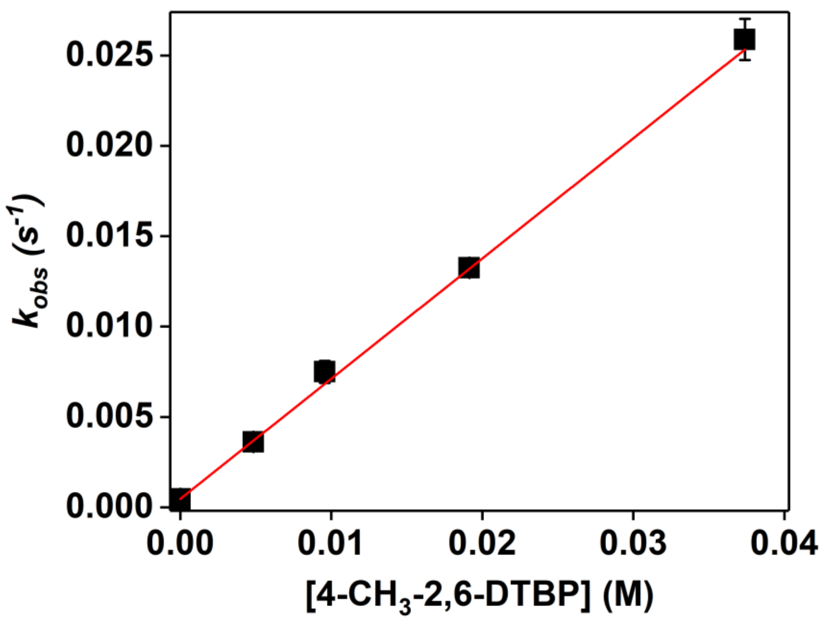

Figure S37 - Plot of k_{obs} vs. [4-CH_3-2,6-DTBP], for the reaction between **2’** and 4-CH_3-2,6-DTBP at -45 °C in DMF. k_2 = 0.67(1) $M^{-1}s^{-1}$.

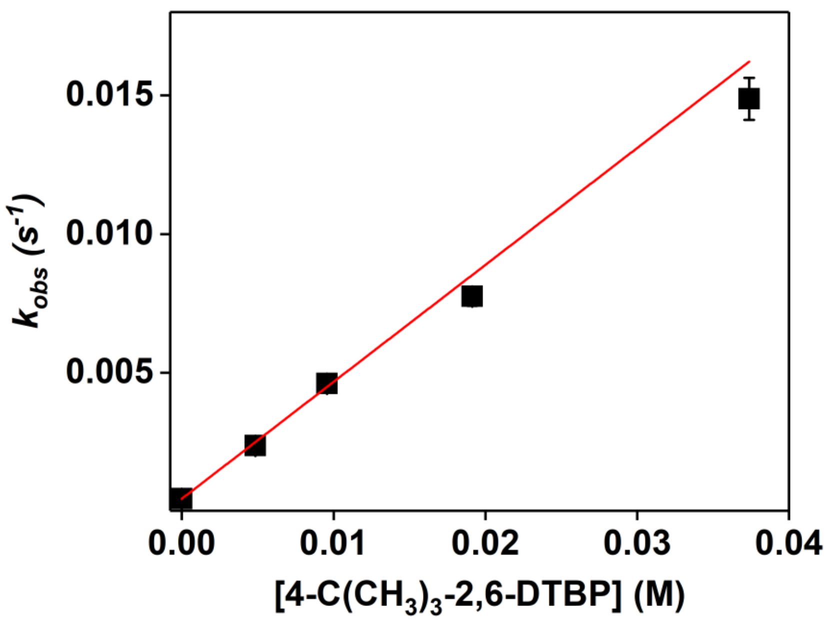

Figure S38 - Plot of k_{obs} vs. [4-$C(CH_3)_3$-2,6-DTBP], for the reaction between **2’** and 4-$C(CH_3)_3$-2,6-DTBP at -45 °C in DMF. k_2 = 0.42(1) $M^{-1}s^{-1}$.

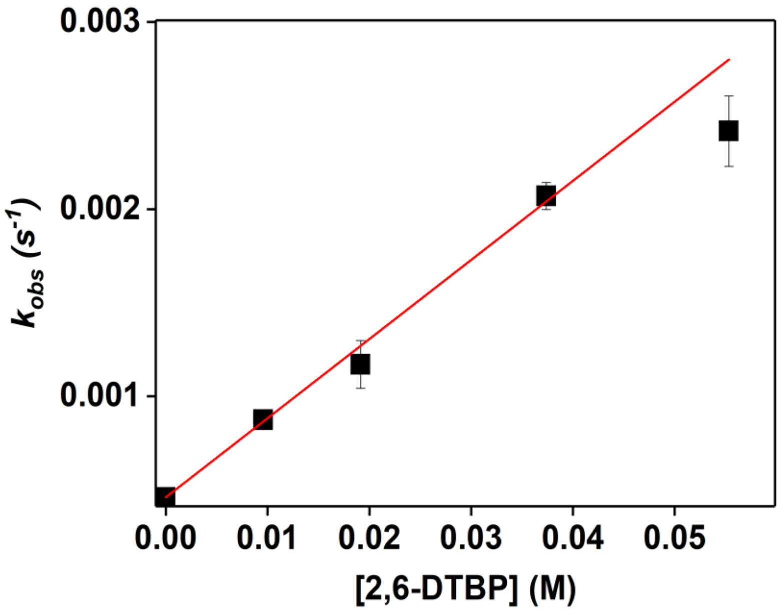

Figure S39 - Plot of k_{obs} vs. [2,6-DTBP], for the reaction between **2’** and 2,6-DTBP at -45 °C in DMF. k_2 = 0.042(2) $M^{-1}s^{-1}$.

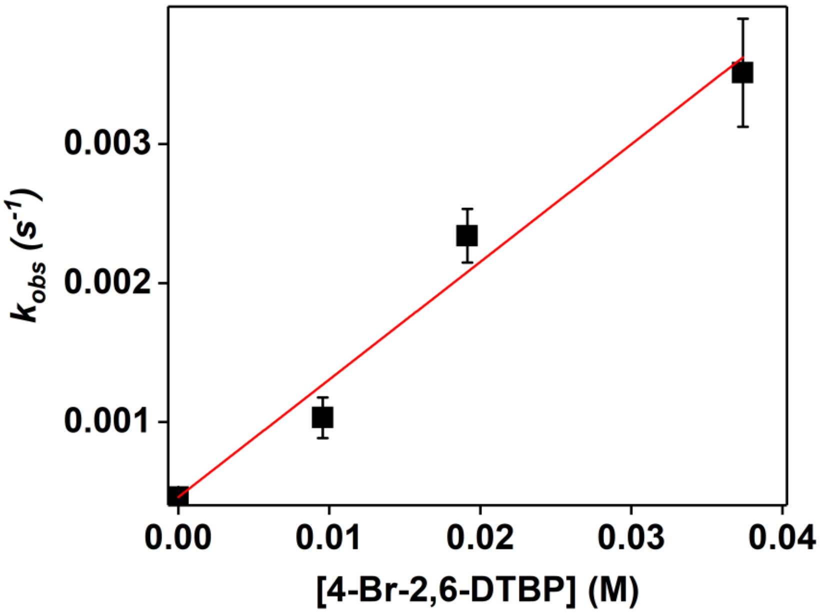

Figure S40- Plot of k_{obs} vs. [4-Br-2,6-DTBP], for the reaction between **2’** and 4-Br-2,6-DTBP at -45 °C in DMF. k_2 = 0.08(1) $M^{-1}s^{-1}$.

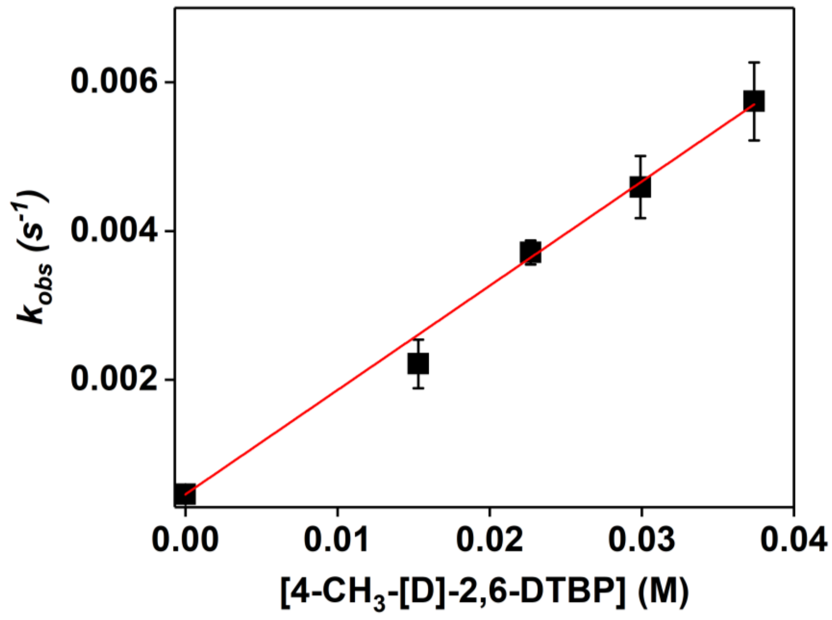

Figure S41 - Plot of k_{obs} vs. [4-CH_3-[D]-2,6-DTBP], for the reaction between **2'** and 4-CH_3-[D]-2,6-DTBP at -45 °C in DMF. k_2 = 0.140(4) $M^{-1}s^{-1}$.

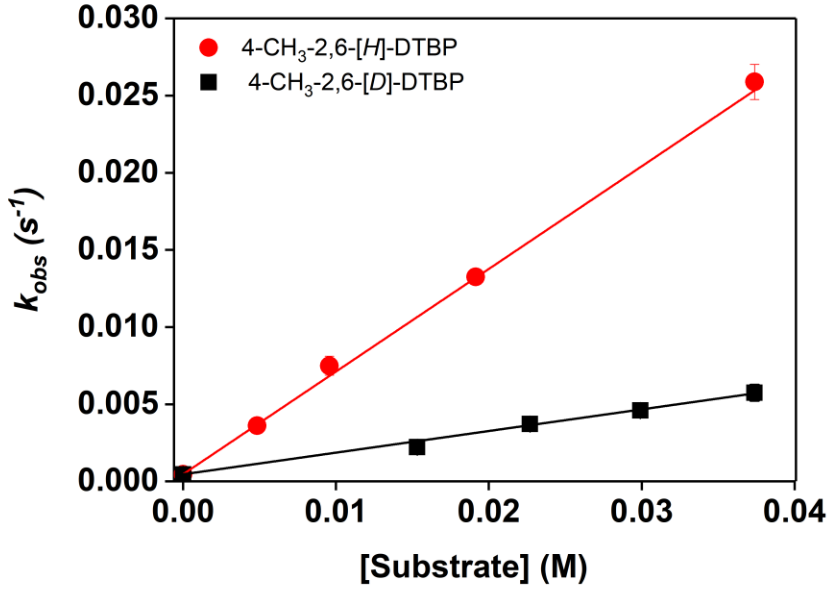

Figure S42 - Plots of k_{obs} versus [substrate] for the reaction of **2'** with 4-CH_3-[*H*]-2,6 DTBP and 4-CH_3-[*D*]-2,6 DTBP, in DMF at -45 °C.

Table S1 – Screened acids and relative outcomes.

	pK_a in DMSO[a]	Outcome
Pyridinium triflate	4.25	quenching
Picolinic acid	8.46	quenching
2-NO_2-benzoic acid	9.37	reaction + shift
2-Br-benzoic acid	10.05	reaction + shift
2,6-dimethyl-benzoic acid	10.40	reaction + shift
3-Cl-benzoic acid	11.04	reaction + shift
Benzoic acid	11.21	reaction + shift
Acetic acid	11.94	reaction
Phenylboronic acid	15.9	No reaction
Boric acid (1st diss.)	16.43	No reaction
H_2O	31.4[5]	No reaction

[a]pK_a in DMSO estimated from the corresponding value in water using the empirical method reported by Knapp et al.[6]

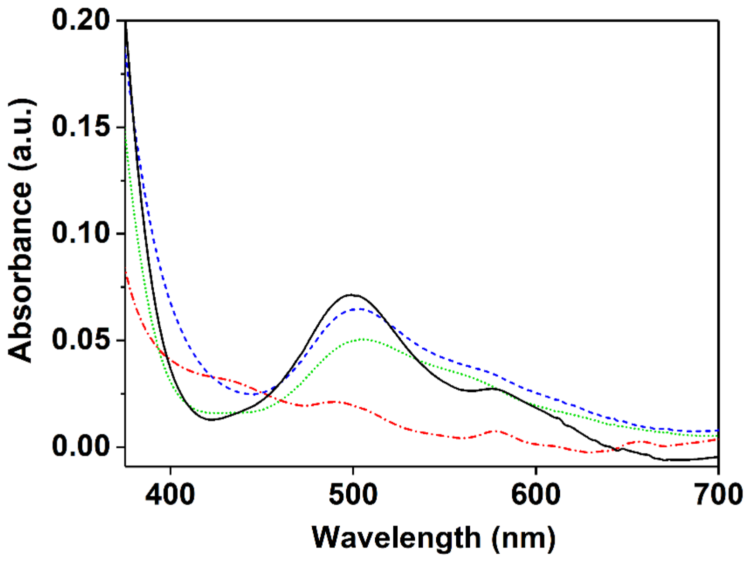

Figure S43– UV-Vis of **1** (502nm feature, black trace) after addition of 1 equiv. (blue dashed trace), 2 equiv, (green dotted trace) and 4 equiv. (red dot-dashed trace) of pyridinium triflate at -45 °C in DMF.

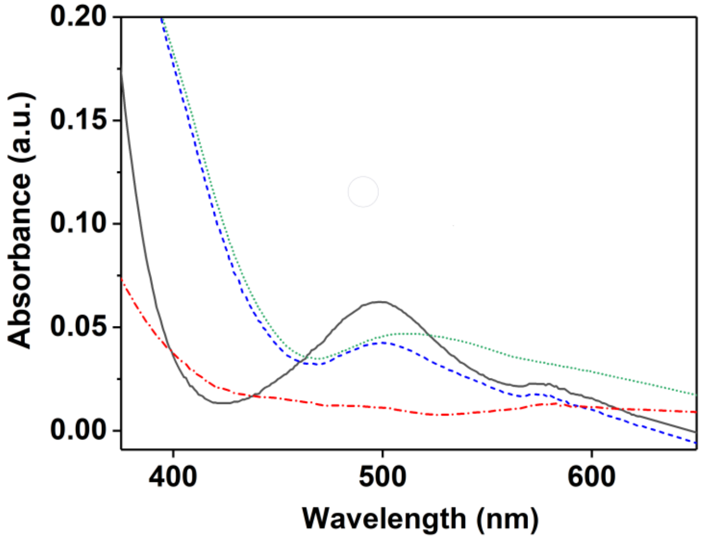

Figure S44 – UV-Vis of **1** (502nm feature, black trace) after addiction of 1 equiv. (blue dashed trace), 2 equiv, (green dotted trace) and 4 equiv. (red dot-dashed trace) of picolinic acid at -45 °C in DMF.

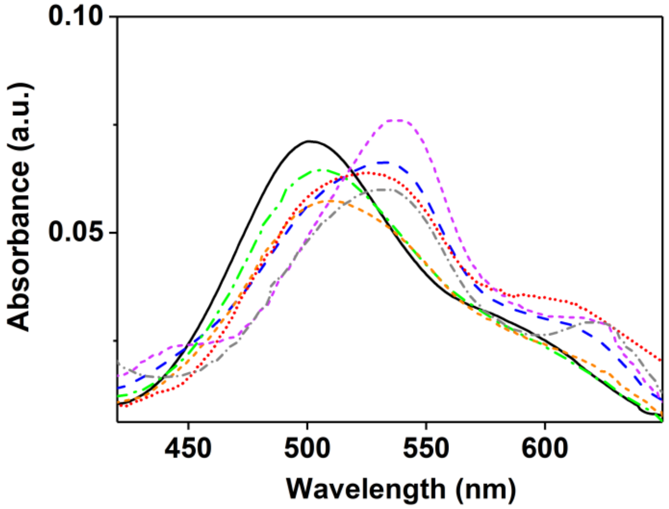

Figure S45 – UV-Vis of **1** (502nm feature, black trace) after addiction of benzoic acid (blue trace), acetic acid, (green trace), 2-Br-benzoic acid (orange trace), 3-Cl-benzoic acid (purple trace), 2-NO_2-benzoic acid (grey trace) and 2,6-dimethyl-benzoic acid (red trace). Each trace corresponds to the addition of 4 equiv. of acid at -45 °C in DMF.

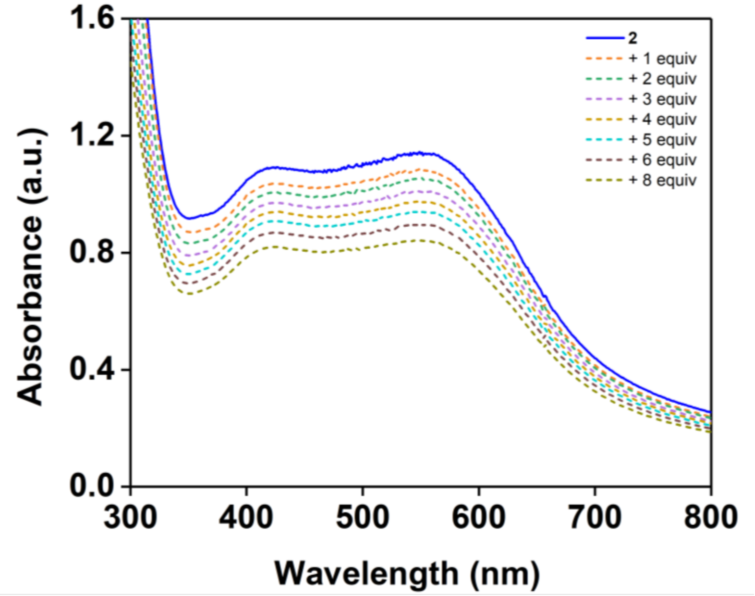

Figure S46 – UV-Vis of **2** (420 nm and 560 nm, feature, blue trace) in presence of different equivalents of 2,6 dimethyl benzoic acid at -45 °C in DMF.

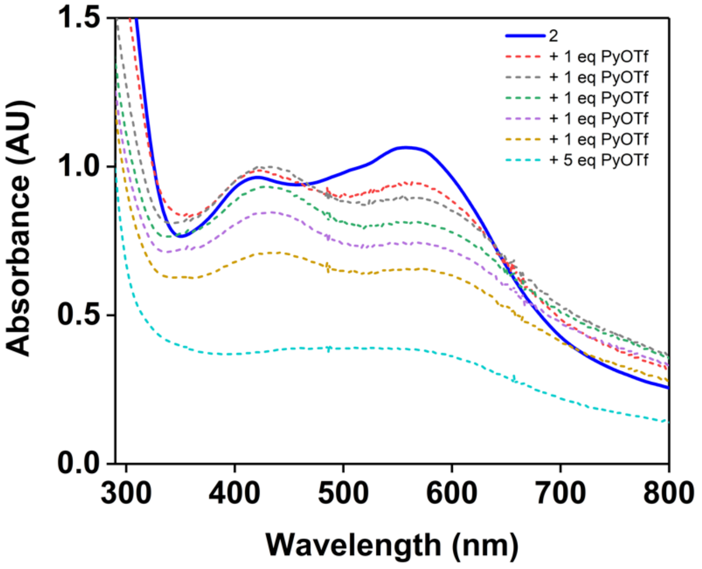

Figure S47 – UV-Vis of **2** (420 nm and 560 nm, feature, blue trace) in presence of different equivalents of pyridinium triflate at -45 °C in DMF.

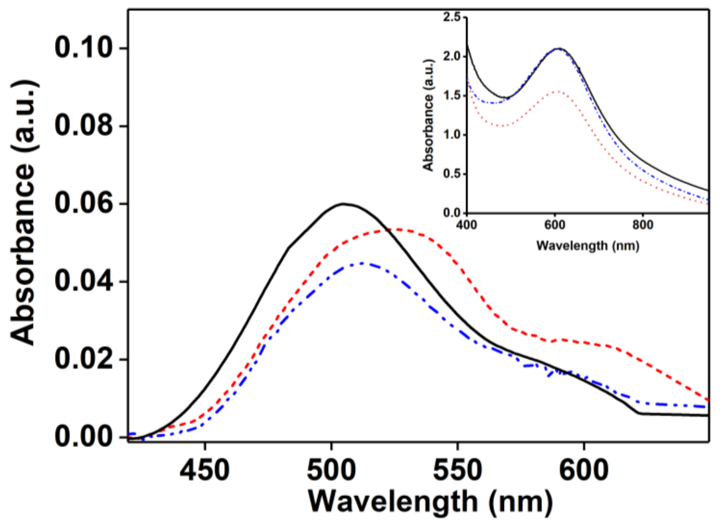

Figure S48 – UV-Vis of **1** (black trace) in the presence of 4 equiv. of 2,6-dimethyl-benzoic acid (red trace) and subsequent addition of 4 equiv. of DBU (blue trace). Inset: comparison of the oxidation yield of **10** (black trace) in the presence of 2,6-dimethyl-benzoic acid (red trace) and subsequent addition of DBU (blue trace).

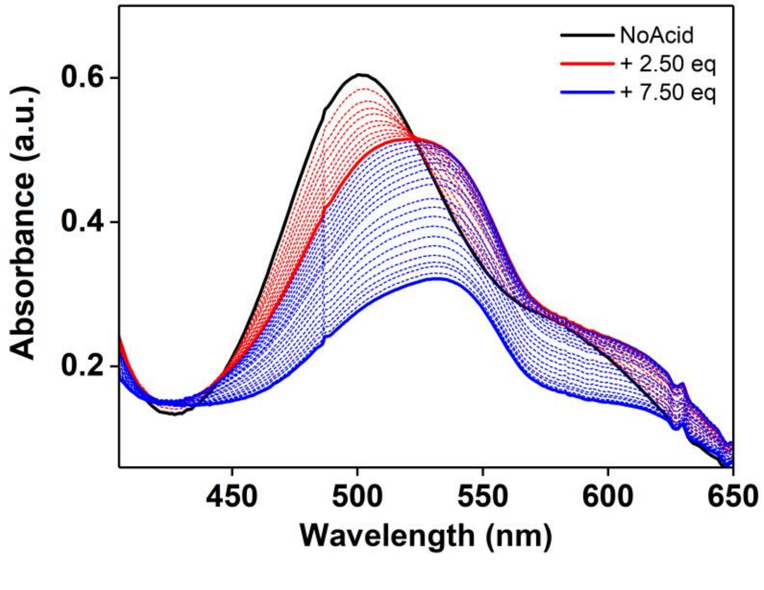

Figure S49 – UV-Vis monitored titration of **1** (black trace) with sub-stoichiometric amount of 2,6 dimethyl benzoic acid at -45˚C in DMF. Each dashed line corresponds to an increment of 0.25 equiv, tilla a maximum of 7.50 equiv.

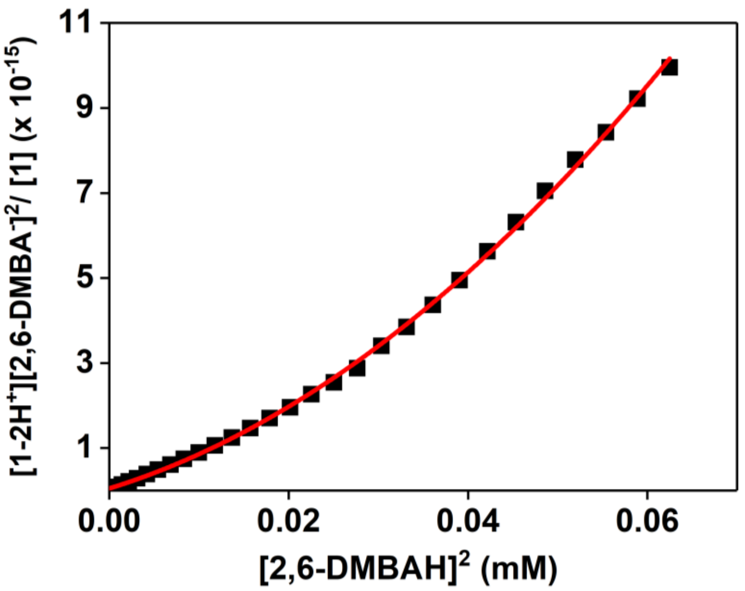

Figure S50. Plot of [2,6-DMBAH]^2 vs. [**1-H**$^+_2$][2,6-DMBA$^-$]^2/[**1**], tentatively fitted with a single protonation event. 2,6-DMBAH = 2,6-dimethyl benzoic acid; 2,6-DMBA$^–$ = 2,6-dimethyl-benzoate.

Table S2 - Parameters related to the series of *para*-X-2,6 di-*tert*-butyl phenols used in the kinetic studies

X	$BDE_{O–H}$ (kcal/mol)[a, 7-10]	$BDFE_{O–H}$ (kcal/mol)[b]	E(V) vs Fc/Fc$^+$[11, 12]	σ^+_p[9]
**$–OCH_3$**	78.3	73.5	0.53	-0.78
**$–CH_2CH_3$**	80.1	75.3	0.87	-0.46[13]
**$–CH_3$**	81	76.2	0.90	-0.31
**$–C(CH_3)_3$**	81.2	76.4	0.93	-0.26
**–H**	82.8	78	1.07	0.00
**–Br**	83.2	78.4	1.10	0.15[14]
**–CN**	84.2	79.4	1.58	0.66
**$–NO_2$**	84.9	80.1	1.70[15]	0.78

[a]The values reported are measured in C_6H_6. [b]The $BDFE_{O–H}$ values are extrapolated from a plot $BDE_{O–H}$ vs $BDFE_{O–H}$ based on the values in C_6H_6 reported by Mayer.[16]

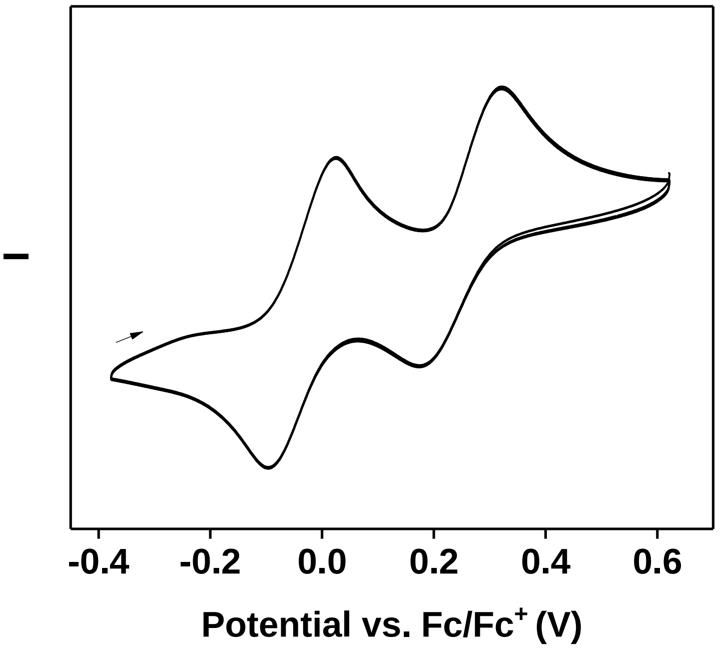

Figure S51 – Steady state cyclic voltammograms of **1**. Conditions: 1.7 mM (DMF), 0.1 M Bu_4NPF_6, scan rate 100 mV s^{-1}, -40 °C.

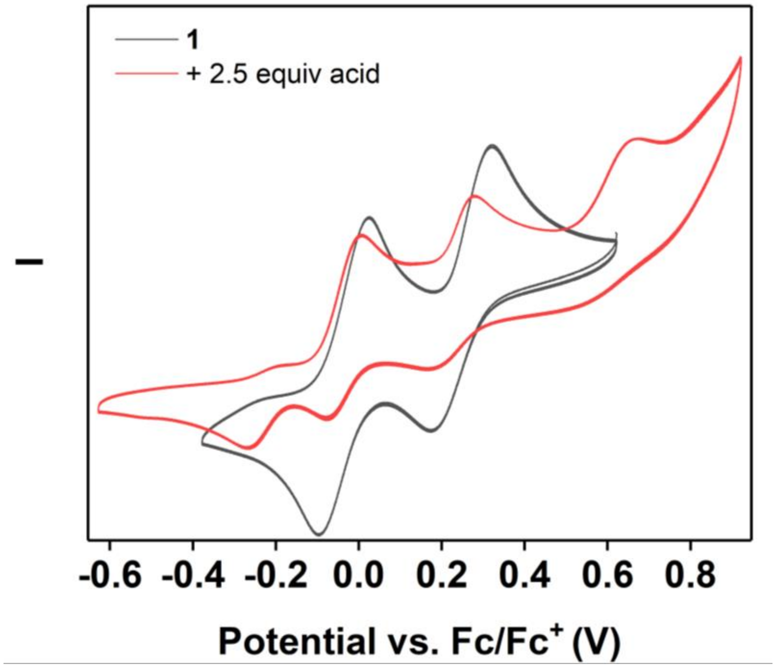

Figure S52 – Steady state cyclic voltammograms of **1** (black trace) and upon addition of 2.5 equivalents of 2,6 dimethyl benzoic acid. Conditions: 1.7 mM (DMF), 0.1 M Bu_4NPF_6, scan rate 100 mV s^{-1}, -40 °C.

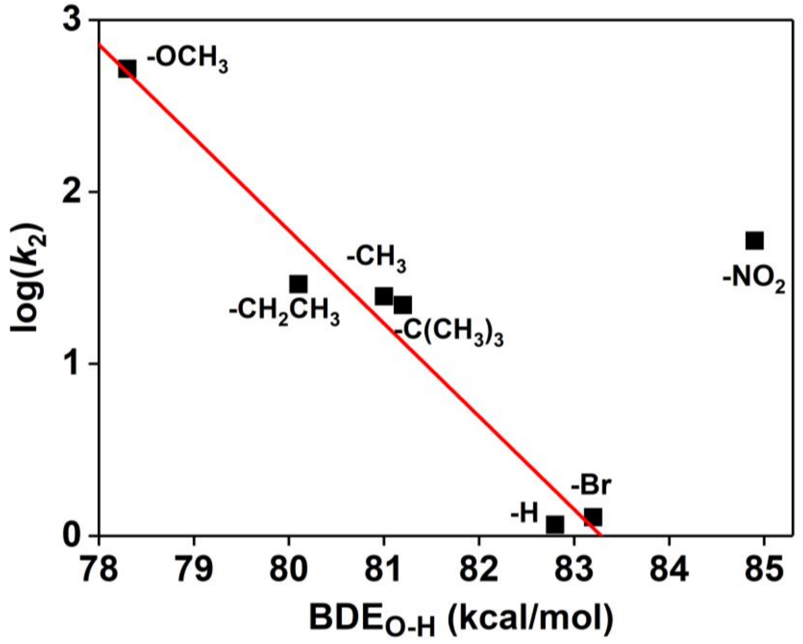

Figure S53 – Plot of log(k_2) against $BDE_{O–H}$ for the reaction of **3** with a series of 4-X-2,6-DTBP in DMF at -45 °C.

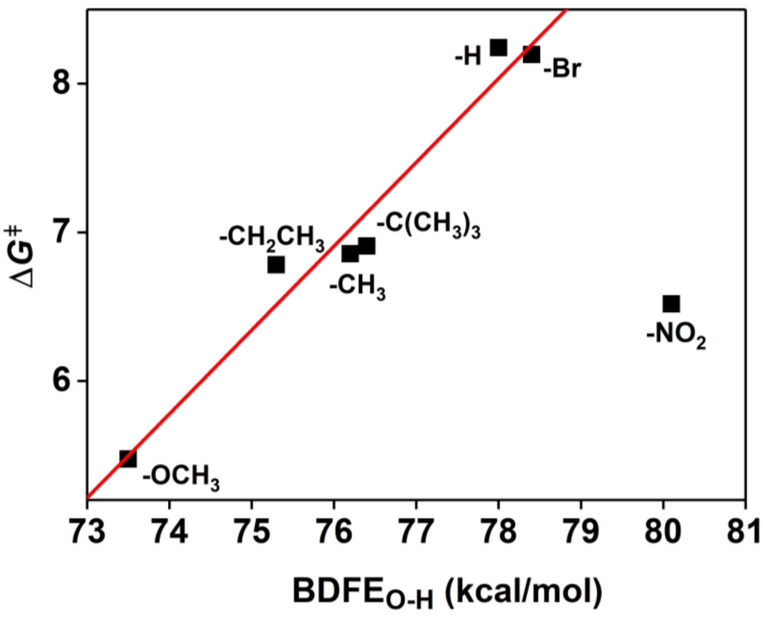

Figure S54 – Evans-Polanyi plot ($\Delta G^{\ddagger}$ vs. $BDFE_{O–H}$) for the reaction of **3** with a series of 4-X-2,6-DTBP in DMF at -45 °C.

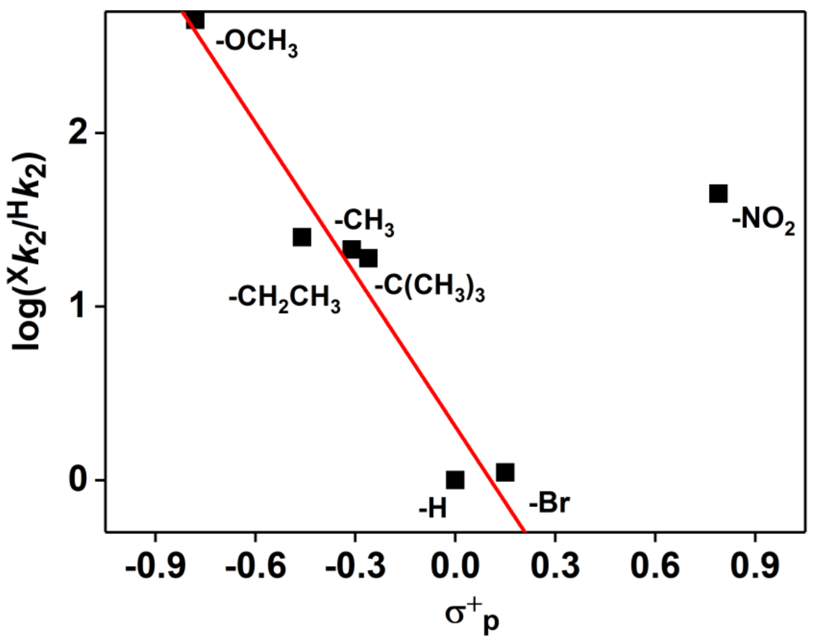

Figure S55 – Hammett correlation plot (log ($^{X}k_2/^{H}k_2$) vs. σ^+_P) for the reaction of **3** with a series of 4-X-2,6-DTBP in DMF at -45 °C.

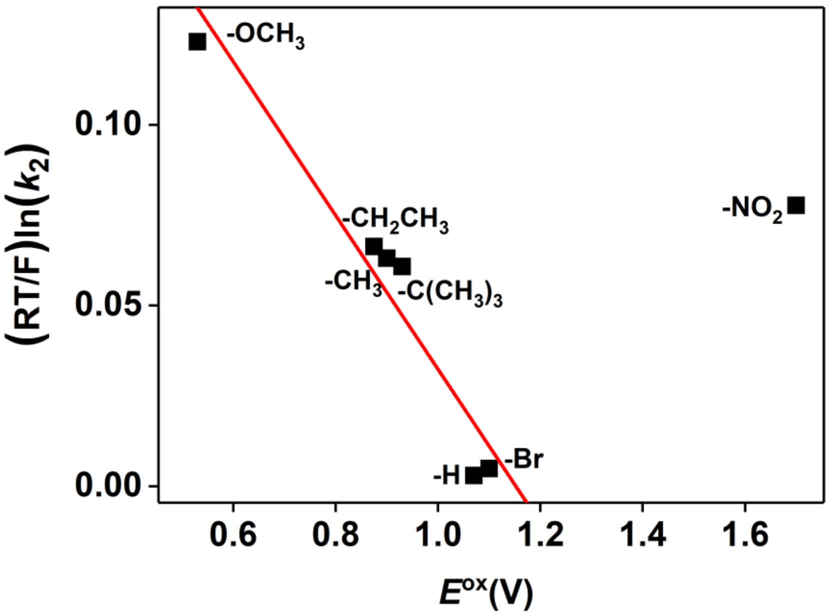

Figure S56 – Marcus correlation plot ((RT/F) ln(k_2) vs. E^{ox}) for the reaction of **3** with a series of of 4-X-2,6-DTBP in DMF at -45 °C.

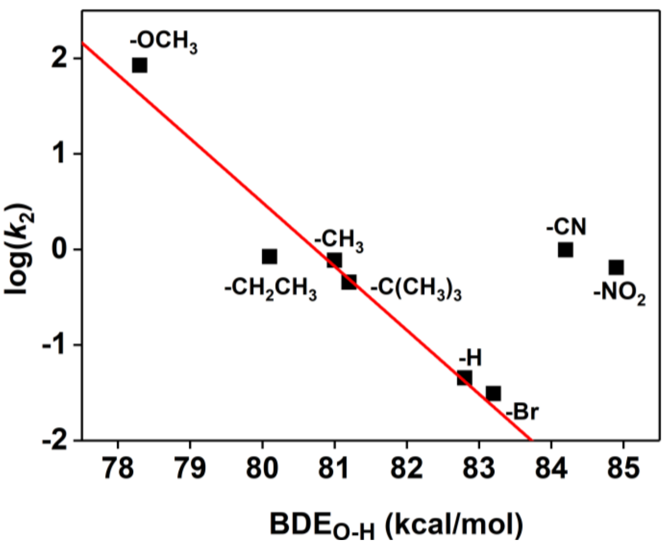

Figure S57– Plot of log(k_2) against $BDE_{O–H}$ for the reaction of **2** with a series of 4-X-2,6-DTBP in DMF at -45 °C.

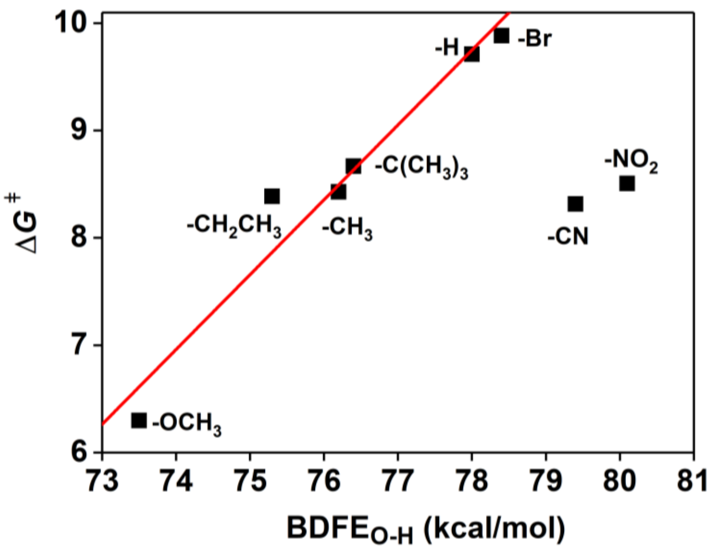

Figure S58 – Evans-Polanyi plot ($\Delta G^‡$ vs. $BDFE_{O–H}$) for the reaction of **2** with a series of 4-X-2,6-DTBP in DMF at -45 °C.

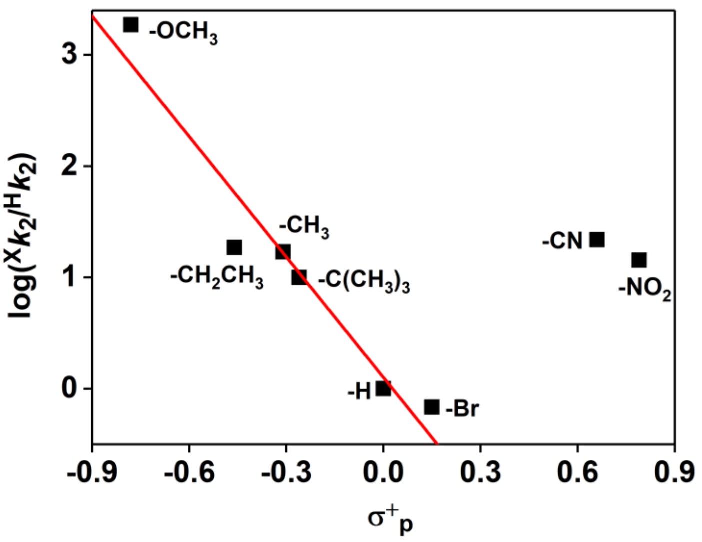

Figure S59 – Hammett correlation plot (log ($^{X}k_2/^{H}k_2$) vs. σ^+) for the reaction of **2** with a series of 4-X-2,6-DTBP in DMF at -45 °C.

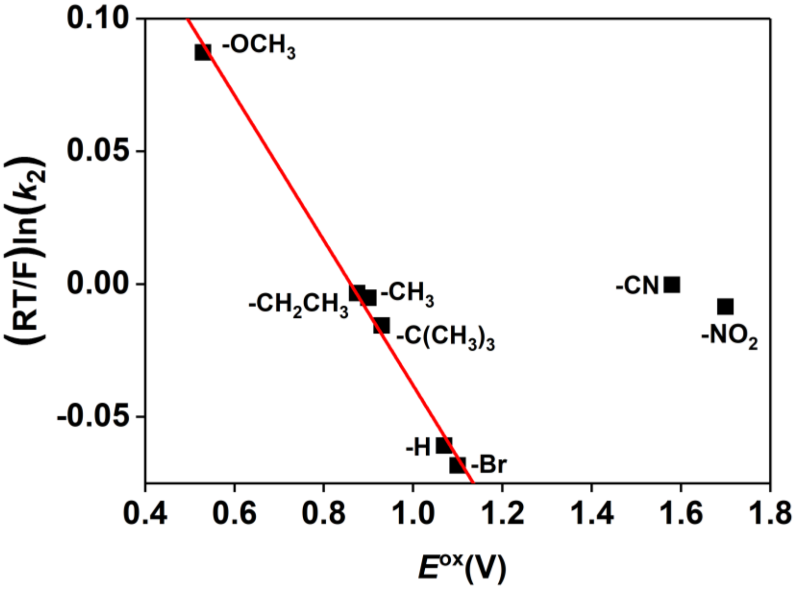

Figure S60 – Marcus correlation plot ((RT/F) ln(k_2) vs. E^{ox}) for the reaction of **2** with a series of 4-X-2,6-DTBP in DMF at -45 °C.

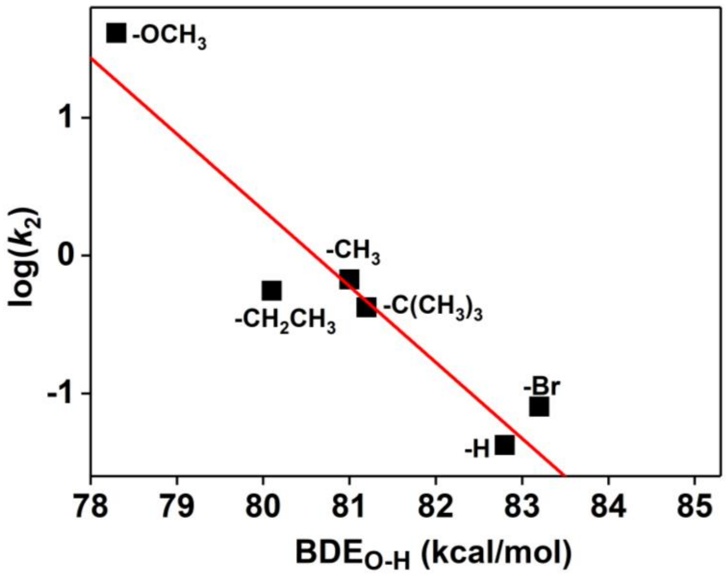

Figure S61 – Plot of log(k_2) against BDE$_{O–H}$ for the reaction of **2’** with a series of 4-X-2,6-DTBP in DMF at -45 °C.

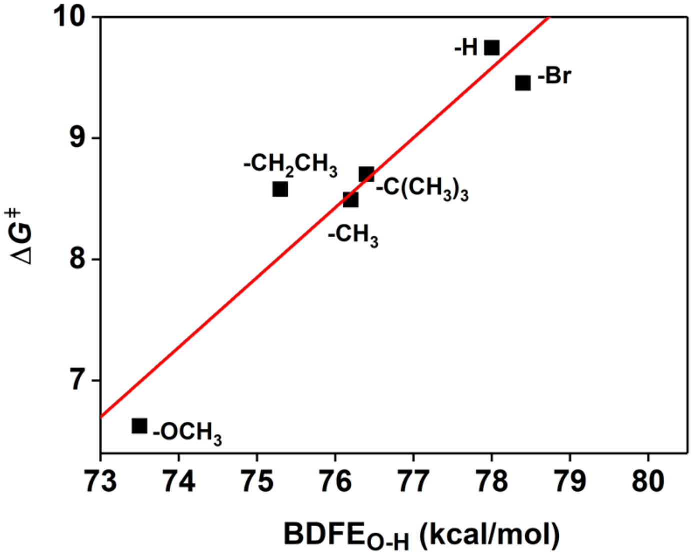

Figure S62 – Evans-Polanyi plot ($\Delta G^{\ddagger}$ vs. BDFE$_{O–H}$) for the reaction of **2’** with a series of 4-X-2,6-DTBP in DMF at -45 °C.

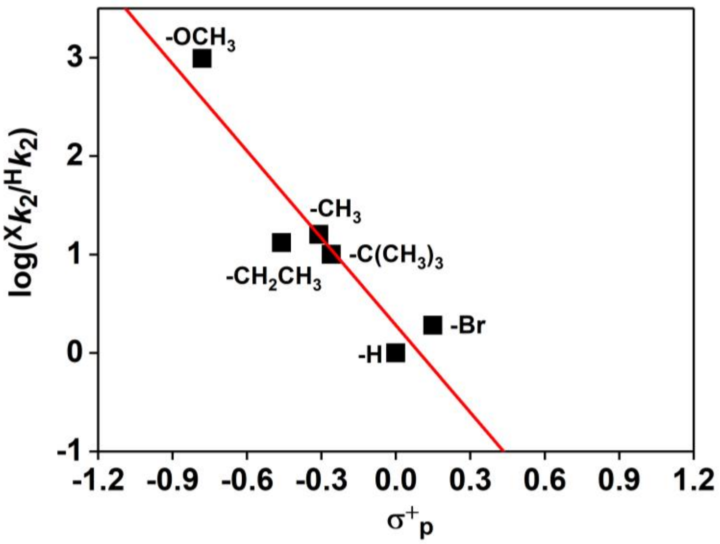

Figure S63 – Hammett correlation plot (log ($^{X}k_2/^{H}k_2$) vs. σ^+) for the reaction of **2'** with a series of 4-X-2,6-DTBP in DMF at -45 °C.

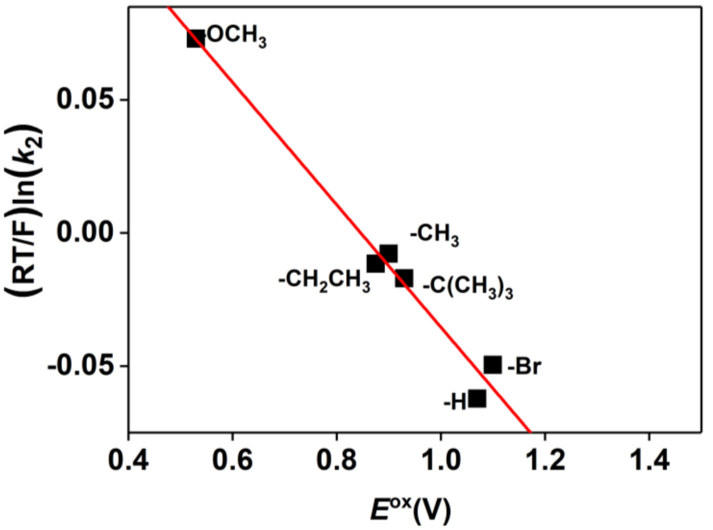

Figure S64 – Marcus correlation plot ((RT/F) ln(k_2) vs. E^{ox}) for the reaction of **2'** with a series of of 4-X-2,6-DTBP in DMF at -45 °C.

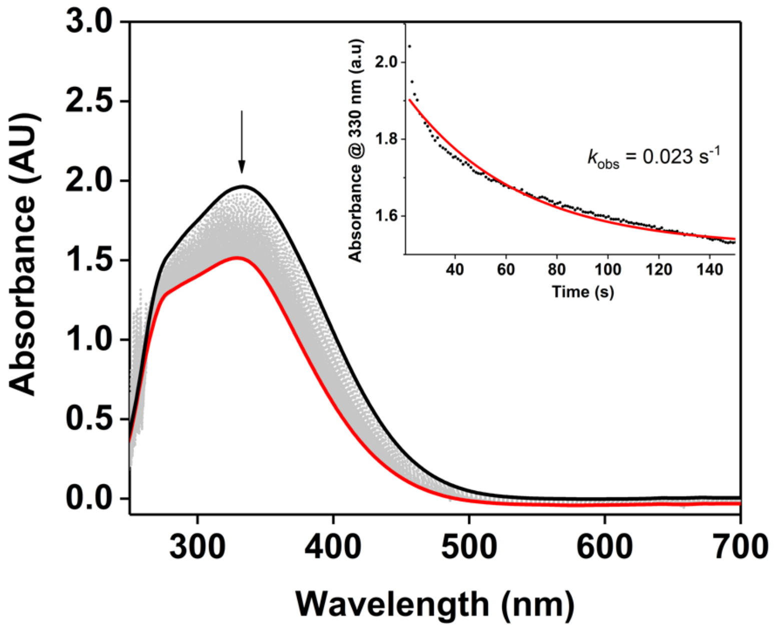

Figure S65 – Electronic absorption spectrum showing the decay of CAN (black trace) over the time at -45 °C in DMF. *Top Inset*. Decay of the λ = 330 nm feature of CAN over the time, resulting in a k_{obs} = 0.023 s^{-1} ($t_{1/2}$ ~ 30 s).

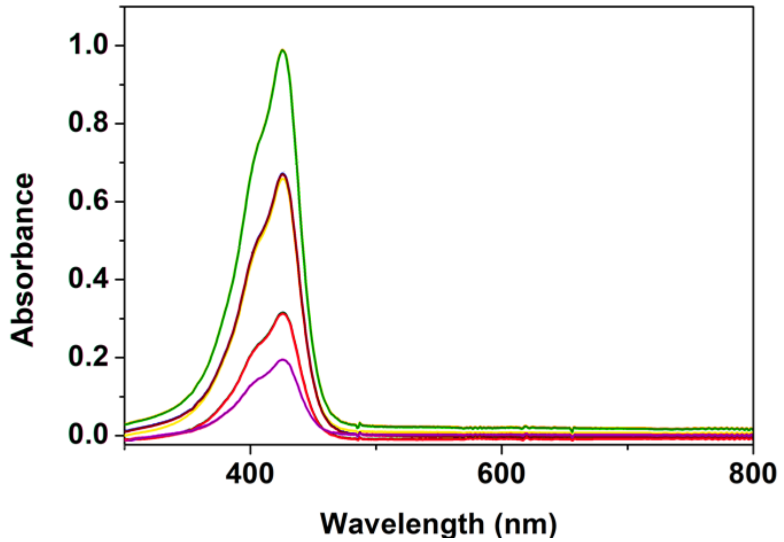

Figure S66. Electronic absorption spectra of DPQ at -45 °C in DMF at different concentration.

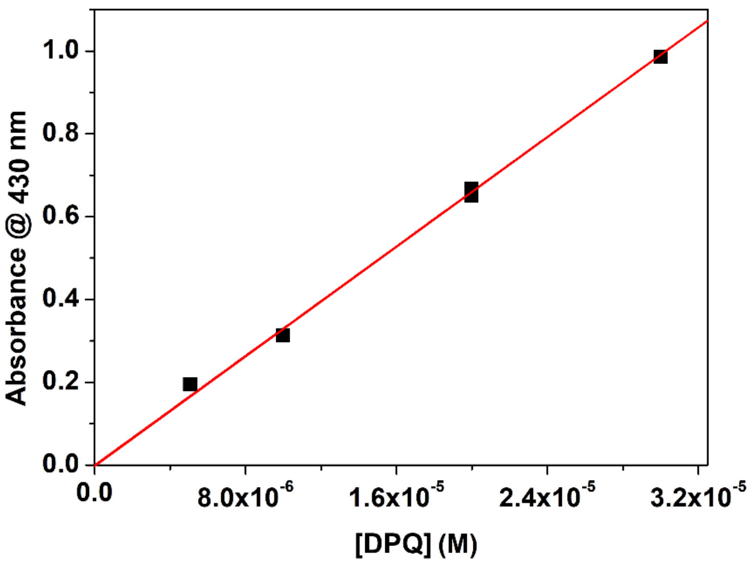

Figure S67. Plot of the absorbance at λ = 430 nm vs. concentration of DPQ at -45°C in DMF, giving the extinction coefficient $\varepsilon_{430\ nm}$ as the slope. ε_{430nm} = 32380 ± 500 $M^{-1}cm^{-1}$.

References

(1) Spedalotto, G.; Gericke, R.; Lovisari, M.; Farquhar, E. R.; Twamley, B.; McDonald, A. R., Preparation and Characterisation of a Bis-μ-Hydroxo-NiIII2 Complex. *Chem. Eur. J.* **2019,** *25* (51), 11983-11990.

(2) Selassie, C. D.; Verma, R. P.; Kapur, S.; Shusterman, A. J.; Hansch, C., QSAR for the cytotoxicity of 2-alkyl or 2,6-dialkyl, 4-X-phenols: the nature of the radical reaction. *J. Chem. Soc. Perk. Trans. 2* **2002,** (6), 1112-1117.

(3) Wittman, J. M.; Hayoun, R.; Kaminsky, W.; Coggins, M. K.; Mayer, J. M., A C-C bonded phenoxyl radical dimer with a zero bond dissociation free energy. *J. Am. Chem. Soc.* **2013,** *135* (35), 12956-9.

(4) Manner, V. W.; DiPasquale, A. G.; Mayer, J. M., Facile Concerted Proton−Electron Transfers in a Ruthenium Terpyridine-4′-Carboxylate Complex with a Long Distance Between the Redox and Basic Sites. *J. Am. Chem. Soc.* **2008,** *130* (23), 7210-7211.

(5) Olmstead, W. N.; Margolin, Z.; Bordwell, F. G., Acidities of water and simple alcohols in dimethyl sulfoxide solution. *J. Org. Chem.* **1980,** *45* (16), 3295-3299.

(6) Rossini, E.; Bochevarov, A. D.; Knapp, E. W., Empirical Conversion of pK a Values between Different Solvents and Interpretation of the Parameters: Application to Water, Acetonitrile, Dimethyl Sulfoxide, and Methanol. *ACS Omega* **2018,** *3* (2), 1653-1662.

(7) Luo, Y.-R., *Handbook of bond dissociation energies in organic compounds*. CRC press: 2002.

(8) Brigati, G.; Lucarini, M.; Mugnaini, V.; Pedulli, G. F., Determination of the substituent effect on the O− H bond dissociation enthalpies of phenolic antioxidants by the EPR radical equilibration technique. *J. Org. Chem.* **2002,** *67* (14), 4828-4832.

(9) Kundu, S.; Chernev, P.; Engelmann, X.; Chung, C. S.; Dau, H.; Bill, E.; England, J.; Nam, W.; Ray, K., A cobalt (II) iminoiodane complex and its scandium adduct: mechanistic promiscuity in hydrogen atom abstraction reactions. *Dalton Trans.* **2016,** *45* (37), 14538-14543.
(10) Lucarini, M.; Pedrielli, P.; Pedulli, G. F.; Cabiddu, S.; Fattuoni, C., Bond Dissociation Energies of O−H Bonds in Substituted Phenols from Equilibration Studies. *J. Org. Chem.* **1996,** *61* (26), 9259-9263.
(11) Lee, J. Y.; Peterson, R. L.; Ohkubo, K.; Garcia-Bosch, I.; Himes, R. A.; Woertink, J.; Moore, C. D.; Solomon, E. I.; Fukuzumi, S.; Karlin, K. D., Mechanistic insights into the oxidation of substituted phenols via hydrogen atom abstraction by a cupric–superoxo complex. *J. Am. Chem. Soc.* **2014,** *136* (28), 9925-9937.
(12) Kundu, S.; Miceli, E.; Farquhar, E. R.; Ray, K., Mechanism of phenol oxidation by heterodinuclear Ni Cu bis (μ-oxo) complexes involving nucleophilic oxo groups. *Dalton Trans.* **2014,** *43* (11), 4264-4267.
(13) The Hammett parameter for this substrate was extrapolated from the linear relationship between the sigma values and BDEs of the other substrates.
(14) Cho, J.; Woo, J.; Eun Han, J.; Kubo, M.; Ogura, T.; Nam, W., Chromium(v)-oxo and chromium(iii)-superoxo complexes bearing a macrocyclic TMC ligand in hydrogen atom abstraction reactions. *Chem. Sci.* **2011,** *2* (10).
(15) Mondal, P.; Pirovano, P.; Das, A.; Farquhar, E. R.; McDonald, A. R., Hydrogen Atom Transfer by a High-Valent Nickel-Chloride Complex. *J. Am. Chem. Soc.* **2018,** *140* (5), 1834-1841.
(16) Warren, J. J.; Tronic, T. A.; Mayer, J. M., Thermochemistry of proton-coupled electron transfer reagents and its implications. *Chem. Rev.* **2010,** *110* (12), 6961-7001.
